# FAM57A (Family with Sequence Similarity 57 Member A) Is a Cell-Density-Regulated Protein and Promotes the Proliferation and Migration of Cervical Cancer Cells

**DOI:** 10.3390/cells11203309

**Published:** 2022-10-21

**Authors:** Dongyun Yang, Tobias D. Strobel, Julia Bulkescher, Claudia Tessmer, Ilse Hofmann, Felix Hoppe-Seyler, Karin Hoppe-Seyler

**Affiliations:** 1 Molecular Therapy of Virus-Associated Cancers, German Cancer Research Center (DKFZ), Im Neuenheimer Feld 242, D-69120 Heidelberg, Germany; 2 Faculty of Biosciences, Heidelberg University, D-69120 Heidelberg, Germany; 3 Genomics and Proteomics Core Facility, Unit Antibodies, German Cancer Research Center (DKFZ), Im Neuenheimer Feld 242, D-69120 Heidelberg, Germany

**Keywords:** cervical cancer, *FAM57A*, human papillomavirus (HPV), hypoxia

## Abstract

The *FAM57A (family with sequence similarity 57 member A)* gene is controversially discussed to possess pro- or anti-tumorigenic potential. Here, we analyze the regulation of cellular FAM57A protein levels and study the functional role of *FAM57A* in HPV-positive cervical cancer cells. We find that FAM57A protein expression strongly depends on cell density, with FAM57A being readily detectable at low cell density, but undetectable at high cell density. This regulation occurs post-transcriptionally and is not mirrored by corresponding changes at the RNA level. We further show that FAM57A protein levels are highly increased in cervical cancer cells cultivated at hypoxia compared to normoxia and provide evidence that *FAM57A* is a hypoxia-responsive gene under control of the α-subunit of the *HIF-1* (hypoxia-inducible factor-1) transcription factor. Yet, the strong relative increase of FAM57A protein levels in hypoxic cells is predominantly cell-density-dependent and occurs post-transcriptionally. Other anti-proliferative effectors besides hypoxia, such as silencing of HPV *E6/E7* oncogene expression in cervical cancer cells, also result in an increase of FAM57A levels compared to untreated cells. Functional analyses reveal that *FAM57A* repression leads to pronounced anti-proliferative as well as anti-migratory effects in cervical cancer cells. Taken together, these results provide insights into the regulation of *FAM57A* protein levels and reveal that they underlie a tight cell-density-dependent control. Moreover, they identify *FAM57A* as a critical determinant for the phenotype of cervical cancer cells, which promotes their proliferation and migration capacities.

## 1. Introduction

The extent of hypoxia (O_2_ tissue concentrations below 1.5–2%) in solid tumors typically correlates with a more aggressive growth behavior and increased therapy resistance [1,2]. This also applies to cervical cancer, a major human cancer with 602,000 new cases and 342,000 cancer deaths in 2020 [3]. We previously showed that cervical cancer cells, which virtually always contain DNA of oncogenic human papillomavirus (HPV) types, induce a state of cellular dormancy under hypoxic conditions (1% O_2_) [4]. This phenotype is characterized by an efficient downregulation of viral *E6/E7* oncogene expression, induction of a reversible cellular growth arrest and increased resistance towards pro-senescent stimuli, including chemotherapeutic agents. These responses of cervical cancer cells could contribute to viral immune evasion, therapy resistance, and tumor recurrence [4,5,6,7]. In order to gain insights into the mechanisms underlying these profound phenotypic alterations, proteome analyses were performed by comparing HPV16-positive SiHa cervical cancer cells cultivated either at hypoxia (1% O_2_) or normoxia (21% O_2_). Notably, the FAM57A (family with sequence similarity 57 member A) protein (alternatively called CT120) emerged as one of the most strongly upregulated proteins in SiHa cells cultivated at hypoxia compared to normoxia [7].

The evolutionary high conservation of the *FAM57A* gene from plants to animals and its broad expression in many human tissues [8] indicates an important biological role. Yet, surprisingly little is known about the regulation of its expression or its cellular function. Four FAM57A protein isoforms have been reported or predicted to result from differential mRNA splicing [8,9]. The longest product of the *FAM57A* gene is a 257 amino acid plasma membrane-associated protein (FAM57A-1 or CT120A), that can interact with the SLC3A2 (solute carrier family 3 member 2) and GGTL3B (gamma-glutamyltranspeptidase-like 3B) proteins and, hence, could play a role in amino acid transport and glutathione metabolism [8].

FAM57A has also been linked to human carcinogenesis and both pro- and anti-tumorigenic activities have been described. For lung, liver, and head and neck cancers, *FAM57A* expression was reported to be increased in tumors compared to the corresponding normal tissues [10,11,12,13,14]. Interference with *FAM57A* expression in cell lines derived from lung and liver cancers acted anti-proliferative [12,13,14], suggesting a pro-tumorigenic role for FAM57A in these tumor entities. In contrast, *FAM57A* was reported to be a protective gene in prostate cancer in that *FAM57A* transcript levels were lower in tumorous than in normal prostate tissue, a decrease of *FAM57A* transcripts correlated with increased prostate cancer grading, and *FAM57A* silencing weakly enhanced the viability of LnCAP prostate cancer cells [15,16]. These latter findings, however, were challenged by a study which correlated increased *FAM57A* transcript levels with unfavorable clinical parameters in prostate cancer patients and reported anti-proliferative effects of *FAM57A* silencing in 22Rv1 prostate cancer cells [17].

In the present work, we analyze the control of FAM57A expression and assess its functional role in cervical cancer cells. We uncover that FAM57A protein levels underlie a striking cell-density-dependent regulation in both HPV-positive and HPV-negative cells. We further show that hypoxia leads to a modest increase of *FAM57A* transcript levels, which is HIF-1α-dependent. However, the profound increase of FAM57A concentrations in cervical cancer cells cultured under hypoxia compared to normoxia occurs mainly at the post-transcriptional level and is a consequence of the decreased cell density resulting from the anti-proliferative effects of hypoxia. Furthermore, functional analyses reveal that blocking FAM57A expression exerts growth inhibitory effects in cervical cancer cells, which are associated with the downregulation of pro-proliferative signaling cascades. In addition, silencing of *FAM57A* expression reduces the migration capacity of cervical cancer cells. Overall, these investigations provide surprising new insights into the regulation of FAM57A and identify FAM57A as an important determinant of the proliferation and migration capacities of cervical cancer cells.

## 2. Materials and Methods

### 2.1. Cell Culture and Treatments

HPV16-positive SiHa (RRID: CVCL_0032) and CaSki (RRID: CVCL_1100), HPV18-positive HeLa cervical cancer cells (RRID: CVCL_0030), as well as HPV-negative HaCaT (RRID: CVCL_0038) skin keratinocytes were obtained from the German Cancer Research Center (DKFZ) tumor bank (Heidelberg, Germany). Due to the genetic heterogeneity of HeLa cells, we also included in key experiments the analysis of two different HeLa laboratory strains (HeLa-1 and HeLa-2), which show differences in the expression of certain cancer-related proteins, such as Smad4 or Dickkopf-1 [18,19]. All analyzed cell lines were authenticated by single-nucleotide polymorphism (SNP) profiling (Multiplexion GmbH, Heidelberg, Germany) and were validated to be free of mycoplasma contamination. Cells were cultivated in Dulbecco’s minimal essential medium (DMEM, Gibco, Thermo Fisher Scientific, Waltham, MA, USA) containing 1 g/L (5.5 mM) glucose, 10% fetal bovine serum (FBS, PAN-Biotech, Aidenbach, Germany), 100 U/mL penicillin, 100 µg/mL streptomycin, and 2 mM L-glutamine (all from Sigma-Aldrich, St. Louis, MO, USA) at 37 °C, 5% CO_2_, and 21% O_2_ (normoxia) if not stated otherwise. For hypoxia experiments, cells were cultivated at 37 °C, 5% CO_2_, and 1% O_2_ using the InvivO_2_ 400 physiological oxygen workstation (Ruskinn Technology Ltd., Bridgend, UK). HeLa-1-mKate2, HeLa-2-mKate2, and SiHa-mKate2 cells were kept under selection by addition of 1 µg/mL puromycin (Sigma-Aldrich), while CaSki-mKate2 cells were kept in culture with 0.5 µg/mL puromycin.

### 2.2. Immunoblot Analyses

Cells were directly lysed in 200 µL Laemmli buffer (10% glycerol, 1% SDS, 0.02 M Tris, 0.1% Bromphenol blue sodium salt, 0.05 M DTT, pH 6.8) supplemented with 125 units Benzonase^®^ (Millipore, Merck, Darmstadt, Germany) for 5 min at room temperature. Whole lysates were subsequently boiled at 95 °C for 5 min and protein concentrations were determined by using the Nanodrop^®^ ND-1000 spectrophotometer (Thermo Scientific, Thermo Fisher Scientific). Immunoblot analyses were performed as previously described [20] and visualized with enhanced chemiluminescence (ECL) using the ECL^™^ Prime Western Blotting Detection Reagent (GE Healthcare, Buckinghamshire, UK), according to the manufacturer’s instructions. Images were obtained by the Fusion SL Detection System (Vilber Lourmat, Eberhardzell, Germany). Uncropped original blots and quantification of immunoblots can be found in Figure S1.

For FAM57A protein expression analyses, a monoclonal anti-FAM57A antibody (clone 2183) recognizing all four predicted isoforms was generated with the support of the Genomics and Proteomics Core Facility (GPCF) Antibody Unit of the German Cancer Research Center (DKFZ), according to the principles of Köhler and Milstein’s hybridoma technology [21]. In brief, BALB/c mouse immunization was performed with the synthetic peptide antigen TWALRRSQPGWSRTDC (PSL GmbH, Heidelberg, Germany), which was C-terminally conjugated through a cysteine residue to keyhole limpet hemocyanin (KLH). Anti-FAM57A antibody-producing B-lymphocytes isolated from positively reacted mice were fused with Sp2/0 murine myeloma cells (RRID: CVCL_2199) to produce hybridoma cell clones. The supernatant from validated monoclonal clones was used for immunodetection of FAM57A protein.

Additionally, the following primary antibodies were used: anti-β-Actin (sc-47778), anti-GAPDH (sc-25778), anti-Vinculin (sc-73614) and anti-HIF-2α (sc-46691) from Santa Cruz Biotechnology (Dallas, TX, USA), anti-phospho-AKT (S473) (#4058), anti-phospho-AKT (T308) (#9275), anti-AKT (#9272), anti-phospho-p44/42 ERK1/2 (Thr202/Tyr204) (#9101) and anti-Cyclin D1 (#2978) from Cell Signaling Technology (Boston, MA, USA), anti-α-Tubulin (CP06, Calbiochem, Sigma-Aldrich), anti-Flag (F3165, Sigma-Aldrich), anti-HIF-1α (#610959, BD Pharmingen, San Diego, CA, USA), and anti-HPV16 E7 (NM2, kind gift of Prof. Martin Müller, German Cancer Research Center, Heidelberg, Germany). Applied secondary antibodies were anti-mouse IgG-HRP (111-035-003) and anti-rabbit IgG-HRP (111-035-071) from Jackson ImmunoResearch (Cambridgeshire, UK).

### 2.3. RNA Extraction and Quantitative Real-Time Polymerase Chain Reaction (qRT-PCR)

Total RNA was isolated by using the PureLink^™^ RNA Mini Kit (Invitrogen, Thermo Fisher Scientific) and reverse transcription was performed by applying the ProtoScript^®^ II First Strand cDNA Synthesis Kit (NEB, Ipswich, MA, USA), following the manufacturer’s instructions. Transcript levels were evaluated by qRT-PCR with the 7300 Real Time PCR System (Applied Biosystems, Thermo Fisher Scientific), using the SYBR^™^ Green PCR Master Mix (Applied Biosystems). The sequences of forward (fwd) and reverse (rev) primers for qRT-PCR were as follows: *18S rRNA* fwd: 5′-CATGGCCGTTCTTAGTTGGT-3′; *18S rRNA* rev: 5′-ATGCCAGAGTCTCGTTCGTT-3′; *TMBIM6* fwd: 5′-GTGGTCATGTGTGGCTTCGT-3′; *TMBIM6* rev: 5′-GGAAAGGCTGGATGGTCACT-3′; *FAM57A* fwd: 5′-AGTGTGGCCAAGAGATCAGC-3′; *FAM57A* rev: 5′-GCCATCATTTCACGCTTCCC-3′; *FAM57A-1* fwd: 5′-GTGCCGAACCAGAGACCAGA-3′; *FAM57A-1* rev: 5′-CGACAAAGAAGTCCCCAAGGT-3′; *FAM57A-2* fwd: 5′-CCTCTGTGAATGGTGCCGAA-3′; *FAM57A-2* rev: 5′-GCTGCTTTAGCTGTGCGAC-3′. The primer pair *FAM57A* fwd/rev used for measuring total *FAM57A* transcript levels recognizes sequences present in all four postulated *FAM57A* transcripts. The *FAM57A-1* fwd/rev primer pair specifically recognizes the *FAM57A-1* transcript, while the *FAM57A-2* fwd/rev primer pair targets the *FAM57A-2* transcript. Transcript expression was quantified using the comparative Ct (2^−ΔΔCt^) method [22] and normalized to a reference gene (*18S rRNA* or *TMBIM6* [23]).

### 2.4. Transfections with Plasmids and siRNAs

All plasmid transfections were performed by calcium phosphate co-precipitation [24]. For the overexpression of N-terminally Flag-tagged FAM57A protein isoforms, the cDNAs of *FAM57A* transcript variants (*FAM57A-1*: NM_024792.3; *FAM57A-2*: NM_001318006.2; *FAM57A-3*: NM_001318007.2; *FAM57A-4*: NM_001318008.2) were amplified by RT-PCR from total RNA of HeLa-1 cells and cloned into the pcDNA3-Flag vector. Short hairpin RNAs (shRNAs) were expressed from pSUPER [25] or pCEPsh plasmids as previously described [26]. Synthetic small interfering RNAs (siRNAs) were transfected with DharmaFECT I (Horizon Discovery, Cambridge, UK) or Lipofectamine RNAiMAX (Invitrogen) according to the manufacturer’s instructions, at a final siRNA concentration of 30 nM. The target sequences for the si/shRNAs were as follows: si/shFAM57A-E1: 5′-GCACCGACUGCGUGAUGAU-3′; si/shFAM57A-E5: 5′-GGAAGGCAGUCCGGCUCUU-3′; siFAM57A-1: 5′-CAGGGUUCUGAUUCAGCUA-3′; siHIF1A-1: 5′-CUAACUGGACACAGUGUGU-3′; siHIF1A-2: 5′-CUGAUGACCAGCAACUUGA-3′; siHIF2A-1: 5′-CAGCAUCUUUGAUAGCAGU-3′; siHIF2A-2: 5′-GCGACAGCUGGAGUAUGAA-3′, si/shNeg: 5′-UACGACCGGUCUAUCGUAG-3′. The efficiencies and specificities of the HIF-1α- and HIF-2α-inhibitory siRNAs are validated in Figure S2. The pool of three different HPV16 *E6/E7*-targeting siRNAs (siE6/E7) as well as si/shCtrl have been described previously [19].

Both siFAM57A-E1 and siFAM57A-E5, which target all four transcripts encoding the predicted FAM57A isoforms, were pooled at equimolar concentrations (referred to as siFAM57A in the text) to minimize potential off-target effects and were applied for total FAM57A downregulation. The siFAM57A-1 recognizes a sequence in exon 4 of the FAM57A-1-encoding transcript, which is not present in the FAM57A-2-encoding transcript; si/shCtrl and si/shNeg are two different negative controls and contain at least four mismatches to all known human genes.

For immunoblot analyses, siRNA-transfected cells were grown for 48 h to confluency, split and seeded at low cell density (if not stated otherwise), and cultivated for another 24 h before harvesting. For the immunoblot analysis measuring p-AKT (S473), p-AKT (T308), AKT, p-ERK1/2 (p-p44/42), and cyclin D1 protein levels upon FAM57A silencing, cells were transfected two times and harvested after 96 h. For live cell imaging assays, reverse transfection was performed in 96-well plates (#3596, Corning, NY, USA) with Lipofectamine RNAiMAX and 30 nM siRNAs.

### 2.5. Colony Formation Assays (CFAs)

For CFAs, cells were transfected with the respective pCEPsh plasmids, split 48 h later, seeded at low density in 6 cm dishes, and selected for hygromycin B resistance. Cells were fixed and stained with formaldehyde and crystal violet solution (12 mM crystal violet, 29 mM NaCl, 3.7% formaldehyde, 22% ethanol) 10 to 15 days after transfection. The areas occupied by colonies (colony area) were quantified in each dish by using an ImageJ macro (Damir Krunic, Light Microscopy Core Facility, DKFZ, Heidelberg, Germany) [27].

### 2.6. Live Cell Imaging Microscopy

For live cell imaging analyses, cells expressing nuclear mKate2 fluorescent protein (HeLa-1-mKate2, HeLa-2-mKate2, SiHa-mKate2 and CaSki-mKate2) were analyzed, which were generated as described previously [27]. All experiments were performed using the IncuCyte^®^ S3 Live-Cell Analysis System (Sartorius, Göttingen, Germany). For proliferation analyses, 3000 cells per well were seeded and reverse transfected in 96-well plates. Four images per well at 10x magnification were obtained every 8 h. Cell numbers over time were determined by counting red nuclei using the IncuCyte^®^ 2019B Rev2 software (Sartorius). For scratch wound assays, 15,000 HeLa-1-mKate2 cells or 20,000 SiHa-mKate2 cells per well were seeded and reverse transfected in 96-well plates. After 2 days, the confluent cells were treated with 5 µg/mL mitomycin C (Enzo Life Sciences, Lörrach, Germany) for 2 h to keep the cell counts constant during the scratch wound assay and minimize the impact of proliferation [28]. Then, the Incucyte^®^ Woundmaker Tool (Sartorius) was employed to create standardized wounds of 700–800 µm width in all wells simultaneously. The relative wound density was assessed using the built-in algorithms of the IncuCyte^®^ 2019B software (Sartorius). Briefly, the relative wound density (RWD) metric describes the cell density within the wound relative to the cell density of the confluent cell layer outside the wound and thus is expected to yield a more accurate metric to assess cell migration into the wound. According to the manufacturer, the relative wound density is calculated as:

$$\%RWD=100*\frac{w\left(t\right)-w\left(0\right)}{c\left(t\right)-w\left(0\right)}$$

with *w*(*t*) being the cell density as determined by the IncuCyte^®^ 2019B software (Sartorius) within the wound and *c*(*t*) being the cell density in the confluent cell layer outside the wound.

### 2.7. Statistical Analyses

All experiments were performed at least three times with consistent results if not stated otherwise. The data from qRT-PCR analyses are presented as mean values following log_2_ transformation with standard deviations (SD), calculated using Microsoft Excel 2016 (Microsoft Corporation, Washington, USA). Statistical significances were analyzed by one-way ANOVA applying SigmaPlot version 14.0 (Systat Software Inc., San Jose, CA, USA). For statistical analyses of CFAs, colony areas were quantified using ImageJ (see above). Raw values were then normalized to the negative control shNeg and log_2_-transformed. Log_2_-transformed fold-change values were used for statistical testing of significance via one-way ANOVA applying SigmaPlot version 14.0 (Systat Software Inc.). For statistical analyses of the live cell imaging curves, raw values of the endpoints were exported via the Incucyte^®^ 2019B Rev2 Software and tested for significance using a Student’s *t*-test in SigmaPlot version 14.0. Statistical significances are indicated as *p* < 0.05 (*), <0.01 (**), or <0.001 (***).

## 3. Results

### 3.1. FAM57A Protein Levels Are Highly Increased in Cells Cultivated at Hypoxia Compared to Normoxia

We previously observed by proteome analyses that FAM57A protein concentrations are strongly increased in HPV16-positive SiHa cervical cancer cells cultivated at hypoxia (1% O_2_) compared to normoxia (standard cell culture conditions, 21% O_2_) [7]. In order to study the expression and regulation of FAM57A at the protein level, an antibody was generated and validated for its suitability for immunoblot analyses. Whereas the antibody detected the four reported or predicted FAM57A variants upon ectopic overexpression, all investigated cervical cancer cell lines only detectably expressed the FAM57A isoform 1 (FAM57A-1 or CT120A) (Figure S3a,b), as corroborated by isoform-specific RNAi (RNA interference) analysis (Figure S3c,d). For reasons of simplicity, this isoform is referred to as FAM57A in the following.

**Figure 1:**
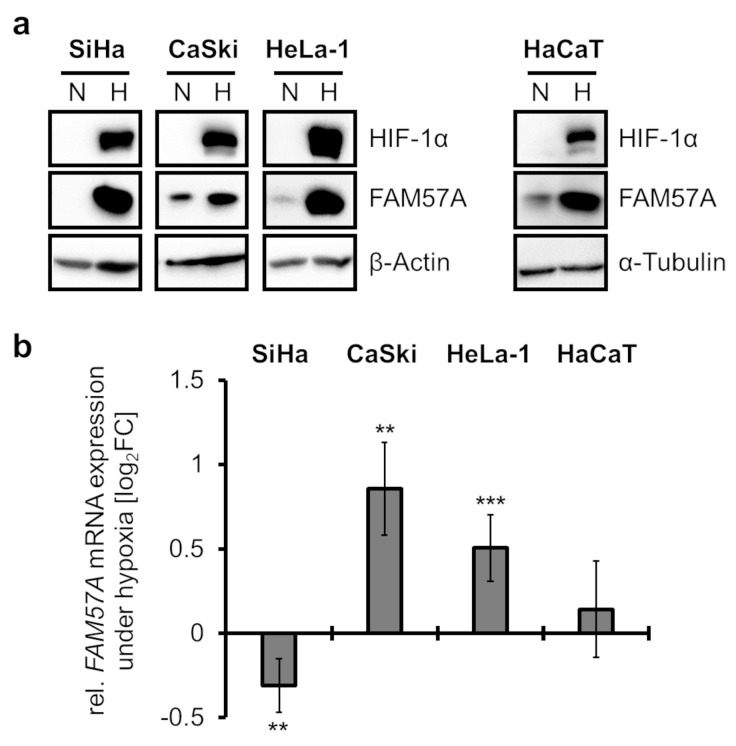
Comparative analyses of FAM57A protein and transcript levels in cells cultivated at normoxia or hypoxia. (**a**) HPV-positive cervical cancer cells (SiHa, CaSki, HeLa-1) and HaCaT keratinocytes were cultivated for 24 h at normoxia (N; 21% O_2_) or hypoxia (H; 1% O_2_) and analyzed by immunoblot for FAM57A protein levels. HIF-1α, hypoxia marker; β-Actin and α-Tubulin, loading controls. (**b**) Corresponding qRT-PCR analyses of *FAM57A* transcript levels under the conditions outlined in subfigure (**a**). Shown are the log_2_-transformed fold changes (log_2_FC) of mean expression in cells cultivated at hypoxia compared to normoxia, with standard deviations from at least 3 independent experiments. Statistically significant differences in cells cultivated at hypoxia compared to normoxia (log_2_FC = 0) are determined by one-way ANOVA. **: *p* < 0.01, ***: *p* < 0.001.

Consistent with the results of the proteome analyses, FAM57A protein levels were substantially higher in immunoblot analyses of SiHa cells cultivated at hypoxia compared to normoxia (Figure 1a). This response was also observed in other cervical cancer cells, such as HPV16-positive CaSki and HPV18-positive HeLa-1 cells (for nomenclature, please refer to the materials and methods section) as well as in HPV-negative HaCaT cells (Figure 1a), which are spontaneously immortalized, non-tumorigenic human keratinocytes [29]. Notably, and in contrast to the strong increase of FAM57A protein amounts in cells cultivated at hypoxia, FAM57A transcript levels were only weakly affected in accompanying qRT-PCR analyses (Figure 1b), indicating that the cellular FAM57A protein concentrations are mainly regulated post-transcriptionally. Collectively, these results confirm the proteome data in SiHa cells, showing that the strong increase of FAM57A concentrations in cells cultivated at hypoxia compared to normoxia is conserved between different cervical cancer cell lines and, furthermore, is not a peculiarity of HPV-positive cancer cells.

### 3.2. FAM57A Protein Levels Are Strongly Dependent on Cell Density

**Figure 2:**
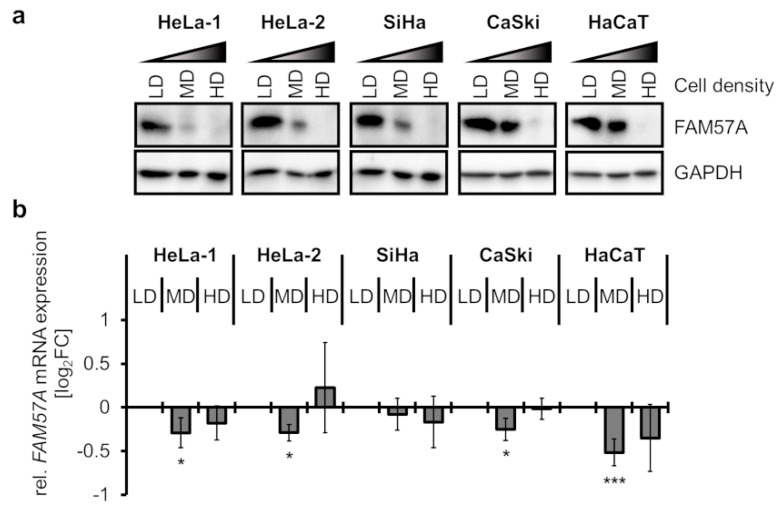
Effects of different cell densities on FAM57A protein and transcript levels. (**a**) Immunoblot analyses of FAM57A protein in cervical cancer cell lines (HeLa-1, HeLa-2, SiHa, CaSki) and HaCaT keratinocytes seeded at low density (LD), medium density (MD), or high density (HD) (please also refer to Figure S4). GAPDH (glyceraldehyde-3-phosphate dehydrogenase), loading control. (**b**) Relative *FAM57A* mRNA concentrations at different cell densities, as assessed by qRT-PCR. Shown are the log_2_FC of mean *FAM57A* transcript levels under MD and HD, relative to LD. Standard deviations from at least 3 independent experiments are indicated. Statistically significant differences compared to the respective cell lines at LD (log_2_FC = 0) are determined by one-way ANOVA. *: *p* < 0.05, ***: *p* < 0.001.

In the course of our experiments, a possible impact of cell density on FAM57A expression was noticed. To systematically test this possibility, we seeded cervical cancer cells and HaCaT keratinocytes at different cell densities (LD, low density; MD, medium density; HD, high density) (Figure S4) and analyzed FAM57A expression under normoxic conditions. Interestingly, FAM57A protein was readily detectable in cells at LD, however, its concentrations were reduced at MD, and FAM57A was virtually undetectable at HD (Figure 2a). In contrast to the pronounced effects on FAM57A protein concentrations, *FAM57A* transcript levels remained largely unaffected at different cell densities (Figure 2b). These results uncover a remarkable cell-density-dependent regulation of cellular FAM57A protein levels, which occurs at the post-transcriptional level and is conserved between different cell types.

### 3.3. FAM57A Protein Levels in Hypoxic Cells Are Primarily a Function of Low Cell Density

**Figure 3:**
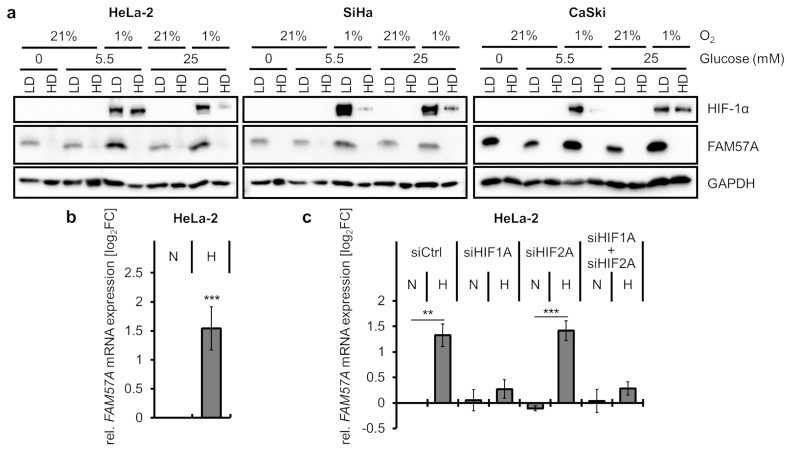
FAM57A expression in relation to cell density, normoxia/hypoxia, and glucose supply. (**a**) Immunoblot analyses of HPV-positive cancer cells seeded at LD or HD. After 24 h, cells were cultivated at normoxic (21% O_2_) or hypoxic (1% O_2_) conditions and under different glucose concentrations, as indicated, for another 24 h before harvesting. HIF-1α, hypoxia marker; GAPDH, loading control. (**b**) qRT-PCR analyses determining the mean (log_2_FC) *FAM57A* transcript levels in HeLa-2 cells cultivated at hypoxia (H; 1% O_2_) compared to normoxia (N; 21% O_2_) (log_2_FC = 0) with standard deviations from 4 independent experiments. Statistically significant differences are determined by one-way ANOVA. ***: *p* < 0.001. (**c**) qRT-PCR analyses measuring *FAM57A* transcript levels following silencing *HIF1A* (siHIF1A) or *HIF2A* (siHIF2A) expression, either alone or in combination. Displayed are the log_2_FC of mean *FAM57A* transcript levels in normoxic (N) and hypoxic (H) cells, relative to control siRNA (siCtrl)-transfected cells cultivated at normoxia (log_2_FC = 0) from 3 independent experiments with standard deviations. Statistically significant differences are determined by one-way ANOVA. **: *p* < 0.01, ***: *p* < 0.001.

Since hypoxia exerts strong anti-proliferative effects in cervical cancer cells [4,7] and in other cell models [30], the question arose whether the high FAM57A levels in hypoxic cells—compared to normoxic cells—may be a function of the growth-inhibitory effects of hypoxia, which results in a reduction of cell density over time, relative to normoxic cells. We therefore analyzed FAM57A levels in cells seeded at LD or HD, which were cultivated either at normoxia or hypoxia. In addition, since the phenotype of hypoxic cervical cancer cells can be substantially influenced by glucose supply [4,7,27], cells were also cultivated at different glucose concentrations of 0 mM, 5.5 mM (corresponding to normal serum glucose concentrations in humans), or 25 mM. We regularly found that FAM57A was readily detectable in cells seeded at LD, but not in cells seeded at HD (Figure 3a). Importantly, this regulation was also observed in cells cultivated at hypoxia (Figure 3a), indicating that the differences in FAM57A expression levels are mainly linked to differences in cell densities but not in O_2_ concentrations. Changes in glucose supply did not markedly influence this regulation (Figure 3a).

In addition, a modest increase of FAM57A protein levels was observed in LD-seeded cells cultivated at hypoxia compared to normoxia, which was most pronounced in HeLa-2 cells (Figure 3a, please compare lane 3 with lane 5 and lane 7 with lane 9). These cells exhibited an approximately 2- to 3-fold upregulation of *FAM57A* transcript levels under hypoxia (Figure 3b). Inspection of the *FAM57A* transcriptional promoter revealed the presence of several potential binding sites (core consensus sequence: A/G-C-G-T-G) [31] for the heterodimeric transcription factors HIF-1 and HIF-2 (hypoxia-inducible factor-1 and -2) (Figure S5), which activate expression of a plethora of genes in response to hypoxia [32]. In order to analyze a potential contribution of the HIF-1α and HIF-2α subunits to the hypoxia-linked *FAM57A* induction, we silenced their expression by RNAi, either individually or in combination (Figure S2). We observed that the hypoxia-induced increase of *FAM57A* transcript levels was counteracted when HIF-1α expression was either blocked alone or in combination with HIF-2α, but not by blocking HIF-2α expression alone (Figure 3c). These results indicate that the strong increase of FAM57A protein levels in cells cultivated at hypoxia compared to normoxia is mainly cell-density-dependent. In addition, HIF-1α modestly increases *FAM57A* transcript levels in hypoxic HeLa-2 cells at LD conditions.

### 3.4. Other Anti-Proliferative Stimuli, Such as HPV E6/E7 Oncogene Silencing, Also Result in a Relative Increase of FAM57A Protein Levels

**Figure 4:**
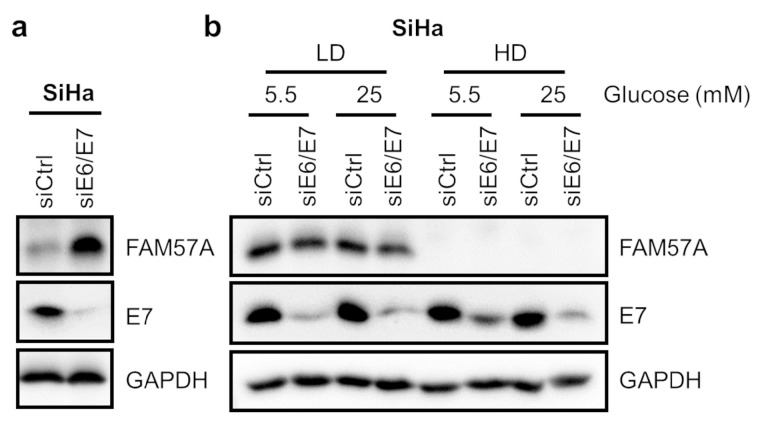
FAM57A expression after HPV *E6/E7* oncogene repression. (**a**) Immunoblot analyses of FAM57A and HPV16 E7 protein levels in SiHa cells after siRNA-mediated *E6/E7* silencing (siE6/E7). siCtrl, control siRNA; GAPDH, representative loading control. (**b**) Immunoblot analyses of FAM57A and HPV16 E7 protein levels in SiHa cells seeded at LD or HD after siRNA-mediated *E6/E7* silencing (siE6/E7), and subsequent cultivation under 5.5 mM or 25 mM glucose for 24 h. siCtrl, control siRNA; GAPDH, representative loading control.

Next, we investigated whether other anti-proliferative stimuli than hypoxia result in a relative increase of *FAM57A* protein levels. In cervical cancer cells, such as HPV16-positive SiHa cells, RNAi-mediated silencing of viral *E6/E7* oncogene expression exerts strong growth-inhibitory effects and results in the rapid induction of cellular senescence [4,33,34]. Notably, compared to control siRNA-transfected cells, which continued to grow and reach high cell densities, *FAM57A* protein levels were increased when *E6/E7* expression was silenced (Figure 4a). This is consistent with the idea that the relative increase of *FAM57A* levels is linked to the anti-proliferative effect of *E6/E7* inhibition, leading to reduced cell densities. We further analyzed cells seeded at LD conditions, under which control siRNA-treated cells did not reach high cell densities during the experiment. Notably, *FAM57A* expression levels were not affected by silencing of HPV *E6/E7* oncogene expression under these conditions (Figure 4b), whereas they are undetectable in cells seeded at HD. As observed for hypoxic conditions (Figure 3a), this regulation of FAM57A protein levels was not altered by differences in glucose supply (5.5 mM vs. 25 mM). Collectively, these data indicate that the relative increase of *FAM57A* concentrations in SiHa cells following HPV *E6/E7* silencing is not a direct, E6/E7-dependent effect on *FAM57A* expression, but a secondary consequence of the anti-proliferative effects of viral oncogene repression.

### 3.5. FAM57A Promotes the Proliferation and Migration of Cervical Cancer Cells

**Figure 5:**
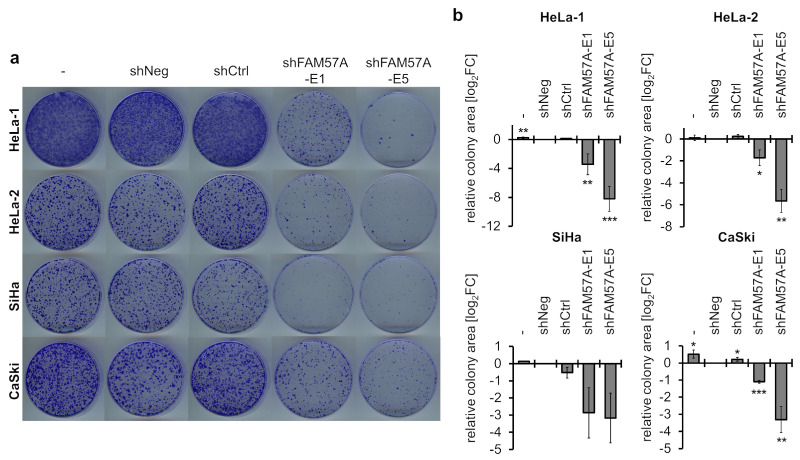
Effects of *FAM57A* silencing on the colony formation capacity of HPV-positive cancer cells. (**a**) Colony formation assays (CFAs) in cervical cancer cell lines expressing *FAM57A*-silencing shRNAs shFAM57A-E1 or shFAM57A-E5 (please also refer to Figure S6a), or two different control shRNAs, shNeg, or shCtrl. (-), empty vector control. Depending on the cell line, cells were grown for 10–15 days and colonies were visualized by staining with crystal violet. (**b**) Quantification of corresponding CFAs (relative colony areas). The presented data are log_2_-transformed and derived from 3 independent CFA analyses of HeLa-1, HeLa-2, and CaSki cells, and from 2 independent CFA analyses of SiHa cells. Standard deviations are indicated. Statistical analyses were performed for HeLa-1, HeLa-2, and CaSki cells. Statistically significant differences are determined relative to shNeg-transfected control cells (log_2_FC = 0) by one-way ANOVA. *: *p* < 0.05, **: *p* < 0.01, ***: *p* < 0.001.

In order to gain insights into the function of FAM57A in cervical cancer cells, we performed colony formation assays (CFAs) upon *FAM57A* silencing, by employing two unrelated *FAM57A*-inhibitory sh (short hairpin) RNAs (Figure S6a). In comparison to cells expressing two different control shRNAs or transfected with the empty vector control, *FAM57A* silencing resulted in a reduction of the colony formation capacity in all investigated cervical cancer cell lines (Figure 5), indicating that *FAM57A* promotes their proliferation.

**Figure 6:**
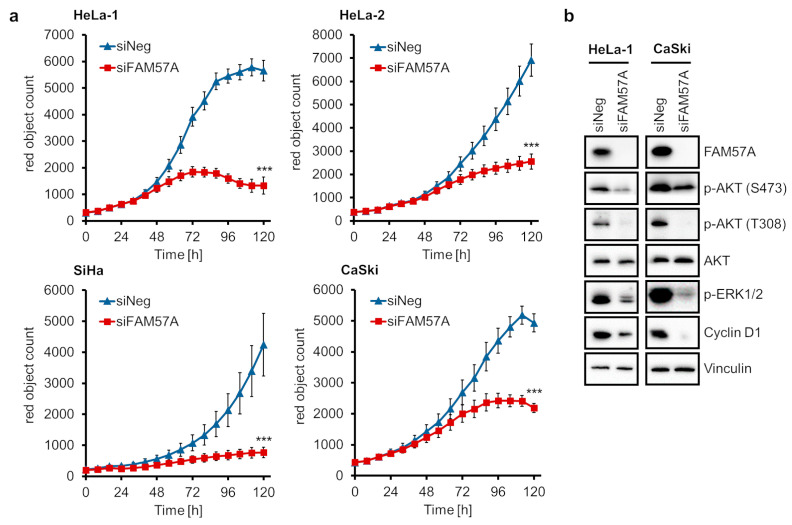
Effects of *FAM57A* silencing on the proliferation of HPV-positive cancer cells. (**a**) Cells were reverse-transfected with the *FAM57A*-inhibitory siRNA pool siFAM57A (consisting of equimolar amounts of siFAM57A-E1 and siFAM57A-E5) in 96-well plates. Cell counts were determined over time for up to 120 h by live cell imaging. Cells express an mKate2-tagged nuclear protein to allow their quantification by the IncuCyte^®^ S3 imaging system. siNeg, control siRNA. Statistically significant differences of the endpoints are analyzed by student’s *t*-test. ***: *p* < 0.001. (**b**) Immunoblot analyses measuring FAM57A, p-AKT (S473), p-AKT (T308), AKT, p-ERK1/2 (p-p44/42), and cyclin D1 protein levels following *FAM57A* silencing in HeLa-1 and CaSki cells. siNeg, control siRNA; vinculin, representative loading control.

Next, we performed proliferation analyses by live cell imaging microscopy. Cells were seeded at LD while being reverse-transfected with a pool of two different *FAM57A*-targeting siRNAs (Figure S6b,c). The resulting growth curves show that *FAM57A* silencing leads to a substantial inhibition of the proliferation of all investigated cervical cancer cell lines when compared to control siRNA-treated cells (Figure 6a), further corroborating the pro-proliferative activity of FAM57A in cervical cancer cells.

Upon ectopic overexpression in NIH3T3 mouse fibroblasts, FAM57A has been reported to possess the potential to stimulate growth-promoting AKT [35]- and ERK [36]-linked signaling [37]. We therefore tested whether endogenous *FAM57A* silencing in cervical cancer cells may affect these signaling cascades. Immunoblot analyses revealed that FAM57A repression is linked to a reduction of phosphorylated p-AKT (S473) and p-AKT (T308) amounts, while not affecting total AKT levels, and with a reduction of phosphorylated ERK1/2 (p-p44/42) and cyclin D1 levels (Figure 6b), the latter being a secondary response factor whose expression is increased by ERK signaling [36]. These findings show that the anti-proliferative effects of FAM57A repression in cervical cancer cells are linked to a reduction of AKT and ERK signaling.

**Figure 7:**
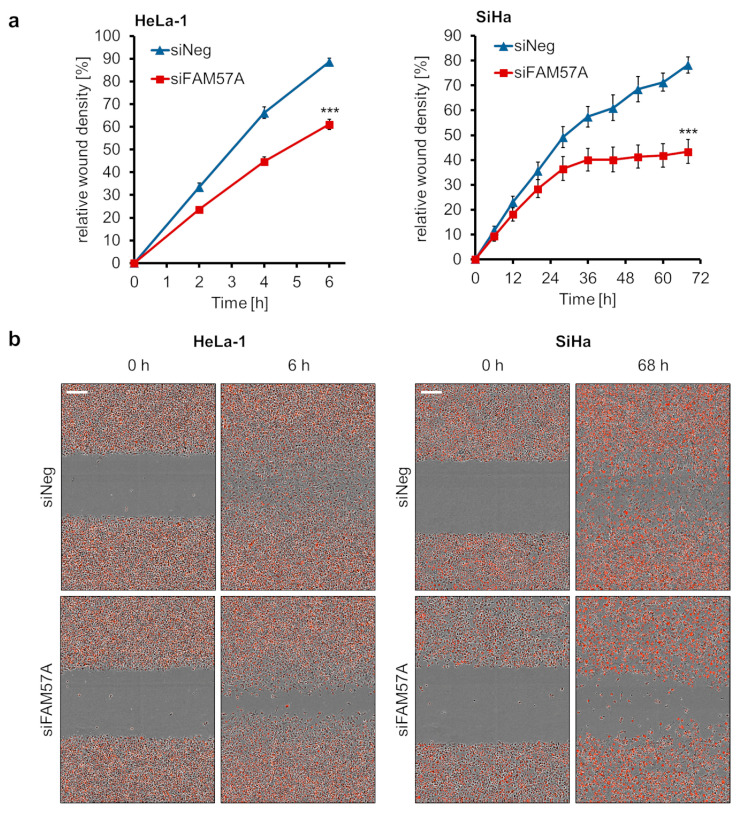
Effects of *FAM57A* silencing on the migration of HPV-positive cancer cells. (**a**) mKate2-labeled HeLa-1 and SiHa cells were reverse transfected with siFAM57A or control siRNA siNeg and seeded into 96-well plates. Following growth to confluency, standardized wounds were introduced into the cell layers by using the IncuCyte Woundmaker tool. The relative wound density was measured over time by live cell imaging, employing the IncuCyte S3 imaging system. Statistically significant differences of the endpoints are analyzed by student’s *t*-test. ***: *p* < 0.001. (**b**) Exemplary microscopic visualization of the cell migration of siFAM57A- or siNeg-transfected mKate2-labeled HeLa-1 and SiHa cells into the wound (time points after scratching: 6 h for HeLa-1, 68 h for SiHa cells). A size marker is indicated in the upper left panel (scale bar: 200 µm).

Finally, we performed scratch wound assays to investigate a possible role of FAM57A for the migration capacity of cervical cancer cells. To this end, cells were seeded and reverse-transfected with *FAM57A*-targeting siRNAs in 96-well plates and, upon reaching confluency, standardized scratches (“wounds”) were introduced into the cell lawn. Live-cell imaging analyses were performed to measure changes in the relative wound density (for details, please refer to the Materials and Methods section) over time. They revealed that *FAM57A* silencing led to a reduction in wound closure when compared with control siRNA-transfected cells (Figure 7a,b), indicating that FAM57A also promotes the migration of cervical cancer cells.

## 4. Discussion

As main results, the present study (*i*) uncovers a striking cell-density-dependent regulation of cellular FAM57A protein levels, which occurs post-transcriptionally, (*ii*) provides evidence that *FAM57A* is a hypoxia-responsive gene and (*iii*) reveals that FAM57A is a critical determinant for the phenotype of cervical cancer cells by promoting their proliferation and migration capacities.

Thus far, there are scant data available concerning the regulation of *FAM57A* gene expression. In head and neck cancers, binding of c-Myc to several binding sites (E-boxes) in the *FAM57A* transcriptional promoter was reported, raising the possibility that the *FAM57A* gene is a target for activation by the c-Myc oncoprotein [38]. In addition, silencing expression of the epigenetic regulator EZH2 (enhancer of zeste homolog 2) led to the downregulation of *FAM57A* transcript levels in colon cancer cells in large scale transcriptome analyses [39]. Here, we show that *FAM57A* is a hypoxia-responsive gene, which is regulated by HIF-1α, but not by HIF-2α. This specificity of *FAM57A* regulation for HIF-1α could be explained by observations that HIF-1 and HIF-2 transactivate only partially overlapping gene sets, despite sharing a common consensus DNA-binding motif [31,40]. In line with being a hypoxia-responsive gene, the *FAM57A* promoter carries multiple consensus DNA binding motifs for HIF-1 (“hypoxia response elements”). Our results are further corroborated by two large scale microarray transcriptome studies reporting an approximately 2-fold increase of *FAM57A* transcript levels in hypoxic breast cancer and hepatic stellate cells [41,42]. This corresponds well to the HIF-1α-dependent, approximately 2- to 3-fold increase of *FAM57A* mRNA concentrations that we detected in our studies in hypoxic HeLa cervical cancer cells.

However, whereas our results indicate that *FAM57A* is a hypoxia-responsive gene, they also show that the strong increase of FAM57A protein levels in cells cultivated under hypoxia compared to normoxia is, to the largest part, dependent on the differences in cell densities, resulting from the anti-proliferative effects of hypoxia. This notion is supported by our finding that—in cells cultivated at different cell densities and different O_2_ concentrations—FAM57A protein levels are primarily dependent on LD cell culture conditions and only to a small extent on hypoxia per se. This observation also emphasizes the importance to control in comparative analyses of cells at hypoxia vs. normoxia (or other conditions differentially affecting cellular proliferation) whether “positive hits” may actually be based on secondary effects, which can result from differences in cell growth, cell density, or other confounding variables. In line with these considerations, siRNA-mediated viral *E6/E7* oncogene repression, which exerts strong anti-proliferative effects in HPV-positive cancer cells [4,33,34], was also linked to a substantial increase of FAM57A protein levels when compared to control siRNA-treated cells. Yet, silencing *E6/E7* expression in *FAM57A*-expressing cells cultivated at LD conditions did not affect FAM57A levels. This indicates that the higher *FAM57A* concentrations in siE6/E7- vs. control-siRNA-treated HPV-positive cervical cancer cells are not a direct effect of the *E6/E7* knockdown, but an indirect effect of the reduced cell densities resulting from the anti-proliferative effects of *E6/E7* repression.

There are data indicating that low cell density can crucially affect the phenotype of cancer cells. For example, cancer cells cultivated at low density have been reported to show decreased microRNA biogenesis, which is linked to enhanced cell proliferation [43], and to exhibit increased invasive and metastatic potential [44]. These activities were linked to the activation of the Hippo signaling cascade [43,44] which is more active at low cell density than at high cell density [45,46]. The concomitant increase of FAM57A protein levels and Hippo signaling at low cell density as well as their overlapping stimulatory effects on tumor cell proliferation and migration raise the question whether there is a functional connection between Hippo pathway and FAM57A. This issue is currently under investigation in the laboratory. It will also be important to measure and spatially resolve the distribution of FAM57A protein expression in normal and cancerous tissues, which could further illuminate the role of FAM57A in the cancer microenvironment.

In addition, our observation that FAM57A expression is highly dependent on cell density can be technically challenging for functional studies and may possibly account for the discrepant results from RNAi analyses on the role of FAM57A for the proliferation of prostate cancer cells [15,17]. In specific, the FAM57A protein levels in the cells under investigation can substantially vary between different seeding densities, with FAM57A amounts being strongly diminished or even undetectable in cells cultivated at HD. To circumvent these potential experimental pitfalls for siRNA analyses, we split cells after transfection to carefully adjust the cell density to conditions under which the FAM57A protein is expressed in control-transfected cells.

Our functional studies uncovered that *FAM57A* silencing exerts pronounced anti-proliferative effects both in short-term live-cell imaging analyses and in long-term CFAs, indicating that FAM57A activity is a critical parameter for the proliferation capacity of cervical cancer cells. The growth-inhibitory effects of *FAM57A* silencing were linked to the downregulation of AKT and ERK signaling, which are known to be important pro-proliferative pathways in HPV-positive cancer cells [47,48]. These findings indicate that FAM57A may support the proliferation of cervical cancer cells, at least in part, via activation of these two growth-promoting signaling cascades. This latter notion is further supported by a study in which FAM57A was ectopically overexpressed in NIH3T3 mouse fibroblasts and resulted in increased AKT and ERK signaling [37].

In addition, we found that *FAM57A* silencing not only acts anti-proliferative in cervical cancer cells but also reduces their migration capacity. From these results, the question arises whether the functional inhibition of FAM57A may possess potential for cancer therapy, by blocking both cancer cell proliferation and migration. Studies reporting increased *FAM57A* expression in lung, liver, and head and neck cancers, when compared to the respective normal tissues [10,11,12,13,14], raise the possibility that there may exist a therapeutic window to preferentially target tumor cells. In this context, it is also thinkable that the hypoxia/HIF-1α-linked increase of *FAM57A* transcript levels observed in our study may contribute to their relative elevation in cancers, since cancers are typically more hypoxic [1] and often exhibit higher HIF-1α levels [49] than corresponding normal tissues. Nevertheless, the results of the present study, which reveal a pronounced post-transcriptional regulation of FAM57A expression without detectable changes in *FAM57A* transcript levels, underline the importance that analyses of clinical material should include the investigation of FAM57A protein levels.

Taken together, we reveal that the cellular FAM57A protein levels underlie a profound cell-density-dependent regulation, occurring at the post-transcriptional level. We further show that the *FAM57A* gene is a hypoxia-responsive gene under control of HIF-1α, and that the strong FAM57A expression in cells cultivated under hypoxia compared to normoxia is primarily cell-density-dependent. Functional analyses reveal that FAM57A is an important determinant for the phenotype of HPV-positive cancer cells and substantially promotes their proliferation and migration. These latter findings also provide a basis for future studies to investigate whether targeting FAM57A for functional inhibition may possess potential to serve as a novel strategy for cervical cancer therapy.

## Data Availability

All data generated or analyzed during this study are included in this published article and its Supplementary Information Files.

## Supplementary Materials

The following supporting information can be downloaded at: https://www.mdpi.com/article/10.3390/cells11203309/s1, Figure S1: Original blots and quantification of immunoblots; Figure S2: Immunoblot analyses measuring HIF-1α and HIF-2α protein levels following silencing *HIF1A* (siHIF1A) or *HIF2A* (siHIF2A) expression, either alone or in combination; Figure S3: FAM57A antibody validation and detection of endogenous FAM57A-1 expression in cervical cancer cells; Figure S4: Microscopic visualization of different cell densities before harvesting the cells for measuring FAM57A expression levels by immunoblot analyses (relates to Figure 2); Figure S5: Potential HIF-1 binding sites in the promoter region (nucleotides –1056 to +64) of the *FAM57A* gene; Figure S6: Validation of shRNAs and siRNAs used for *FAM57A* silencing.

## Abbreviations

CFA, colony formation assay; FAM57A, Family with sequence similarity 57 member A; HD, high density; HIF, hypoxia-induced factor; HPV, human papillomavirus; LD, low density; log_2_FC, log_2_-transformed fold change; MD, medium density; qRT-PCR, quantitative real-time polymerase chain reaction; RNAi, RNA interference; shRNA, short hairpin RNA; siRNA, small interfering RNA.
