# Supplementary material for: FAM57A (Family with Sequence Similarity 57 Member A) Is a Cell-Density-Regulated Protein and Promotes the Proliferation and Migration of Cervical Cancer Cells
Source: Cells. 2022 Oct 21;11(20):3309. doi: 10.3390/cells11203309 (PMC9600422; doi:10.3390/cells11203309)
Supplement: Supplementary file 1 [file cells-11-03309-s001.zip › cells-1943028-supplementary.pdf]

# Supplementary Materials

## FAM57A (Family with sequence similarity 57 member A) is a cell density-regulated protein and promotes the proliferation and migration of cervical cancer cells

Dongyun Yang[1], Tobias D. Strobel[1,2], Julia Bulkescher[1], Claudia Tessmer[3], Ilse Hoffmann[3], Felix Hoppe-Seyler[1,*], Karin Hoppe-Seyler[1,*]

[1]Molecular Therapy of Virus-Associated Cancers, German Cancer Research Center (DKFZ), Im Neuenheimer Feld 242, D-69120 Heidelberg, Germany, [2]Faculty of Biosciences, Heidelberg University, D-69120 Heidelberg, [3]Genomics and Proteomics Core Facility, Unit Antibodies, German Cancer Research Center (DKFZ), Im Neuenheimer Feld 242, D-69120 Heidelberg, Germany

Supplementary Figures S1-S6

Related to Figure 1a:

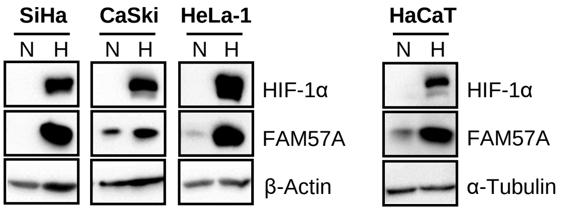


Original blots and quantification of blots corresponding to Figure 1a:

**SiHa**

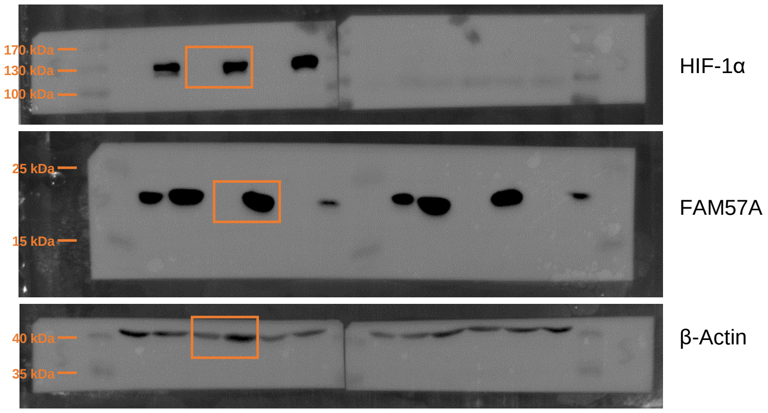


| SiHa | | | |
|---|---|---|---|
| Lane | 1 | 2 | |
| **β-Actin** | 78.057 | **128.453** | raw values |
| **FAM57A** | 1.608 | **170.891** | raw values |
| | 0.015485 | **1** | relative values |
| **HIF-1α** | 0.883 | **182.547** | raw values |
| | 0.007960 | **1** | relative values |

**CaSki**

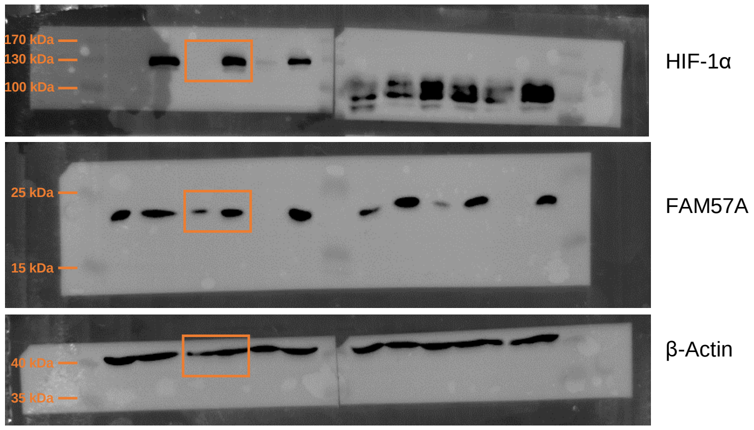


| CaSki | | | |
|---|---|---|---|
| Lane | 1 | 2 | |
| **β-Actin** | 118.130 | **154.991** | raw values |
| **FAM57A** | 70.912 | **153.885** | raw values |
| | 0.604602 | **1** | relative values |
| **HIF-1α** | 3.088 | **206.814** | raw values |
| | 0.01959 | **1** | relative values |

**HeLa-1**

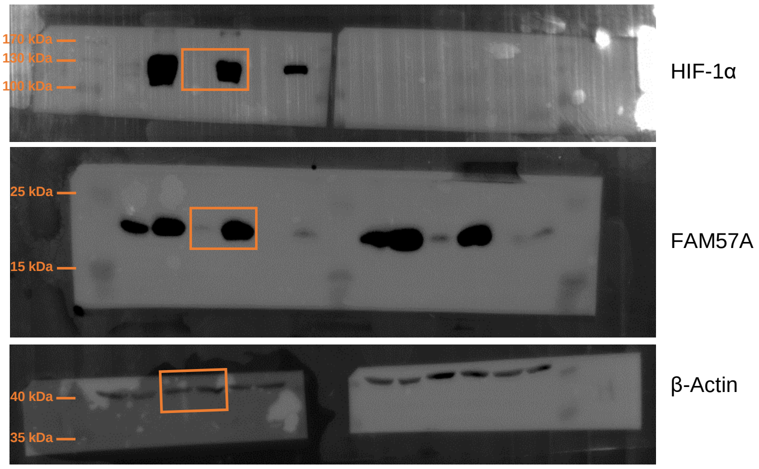


| HeLa-1 | | | |
|---|---|---|---|
| Lane | 1 | 2 | |
| **β-Actin** | 81.973 | **109.305** | raw values |
| **FAM57A** | 20.125 | **185.431** | raw values |
| | 0.144718 | **1** | relative values |
| **HIF-1α** | 5.391 | **192.951** | raw values |
| | 0.037256 | **1** | relative values |

**Figure S1**. Original blots and quantification of immunoblots. Uncropped original immunoblots including molecular weight markers are depicted. Immunoblots were quantified densitometrically with background subtraction and inversion (raw values), and were normalized to loading control and subsequently to the value in blue (relative values). Continued on next pages.

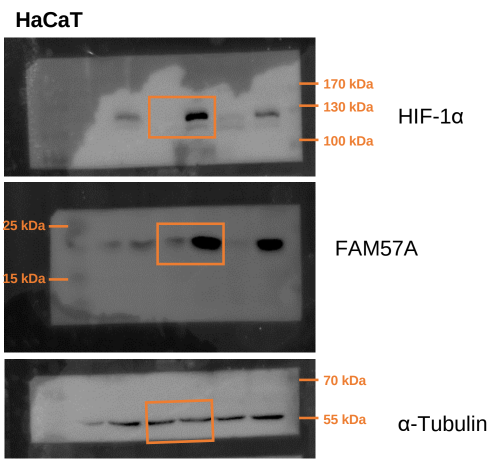


| | HaCaT | | |
|---|---|---|---|
| Lane | 1 | 2 | |
| α-Tubulin | 131.726 | **120.956** | raw values |
| FAM57A | 46.410 | **159.639** | raw values |
| | 0.266949 | **1** | relative values |
| HIF-1α | 0 | **160.714** | raw values |
| | 0 | **1** | relative values |

Related to Figure 2a:

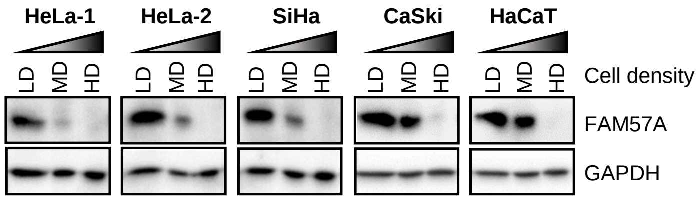


Original blots and quantification of blots corresponding to Figure 2a:

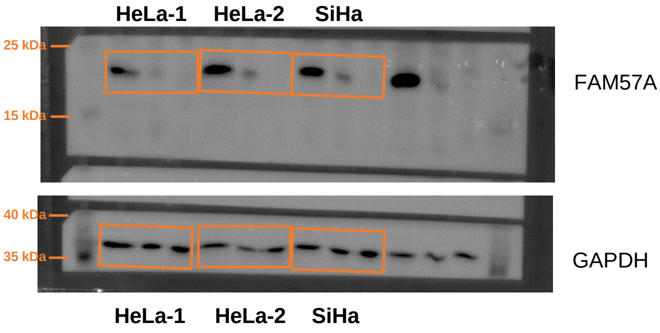


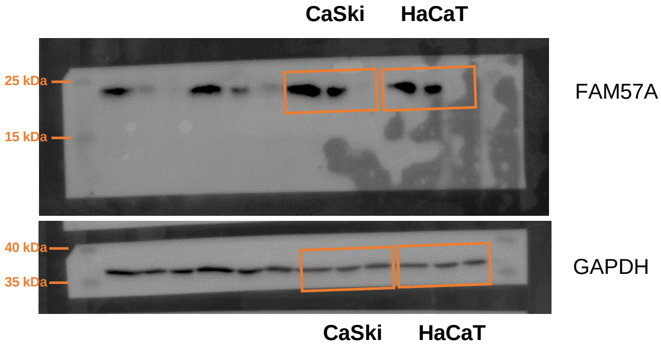


| | HeLa-1 | | | HeLa-2 | | | SiHa | | | |
|---|---|---|---|---|---|---|---|---|---|---|
| Lane | 1 | 2 | 3 | 1 | 2 | 3 | 1 | 2 | 3 | |
| GAPDH | **169.365** | 127.143 | 138.009 | **129.886** | 87.130 | 112.965 | **162.34** | 148.830 | 173.932 | raw values |
| FAM57A | **131.580** | 27.730 | 9.924 | **166.187** | 49.407 | 1.444 | **157.294** | 53.556 | 2.986 | raw values |
| | **1** | 0.280732 | 0.092558 | **1** | 0.443186 | 0.009991 | **1** | 0.371391 | 0.017718 | relative values |

| | CaSki | | | HaCaT | | | |
|---|---|---|---|---|---|---|---|
| Lane | 1 | 2 | 3 | 1 | 2 | 3 | |
| GAPDH | **133.659** | 128.594 | 141.471 | **124.573** | 117.447 | 118.540 | raw values |
| FAM57A | **191.790** | 132.016 | 12.749 | **147.683** | 101.418 | 2.158 | raw values |
| | **1** | 0.715448 | 0.062803 | **1** | 0.728394 | 0.01536 | relative values |

**Figure S1** continued.

Related to Figure 3a:

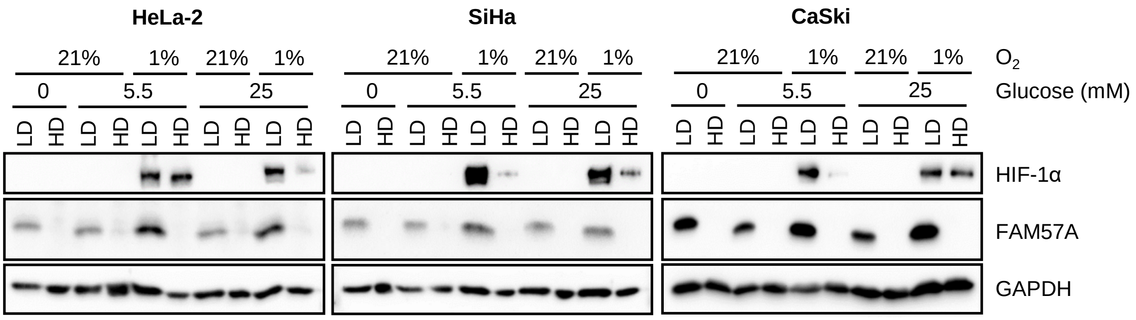


Original blots and quantification of blots corresponding to Figure 3a:

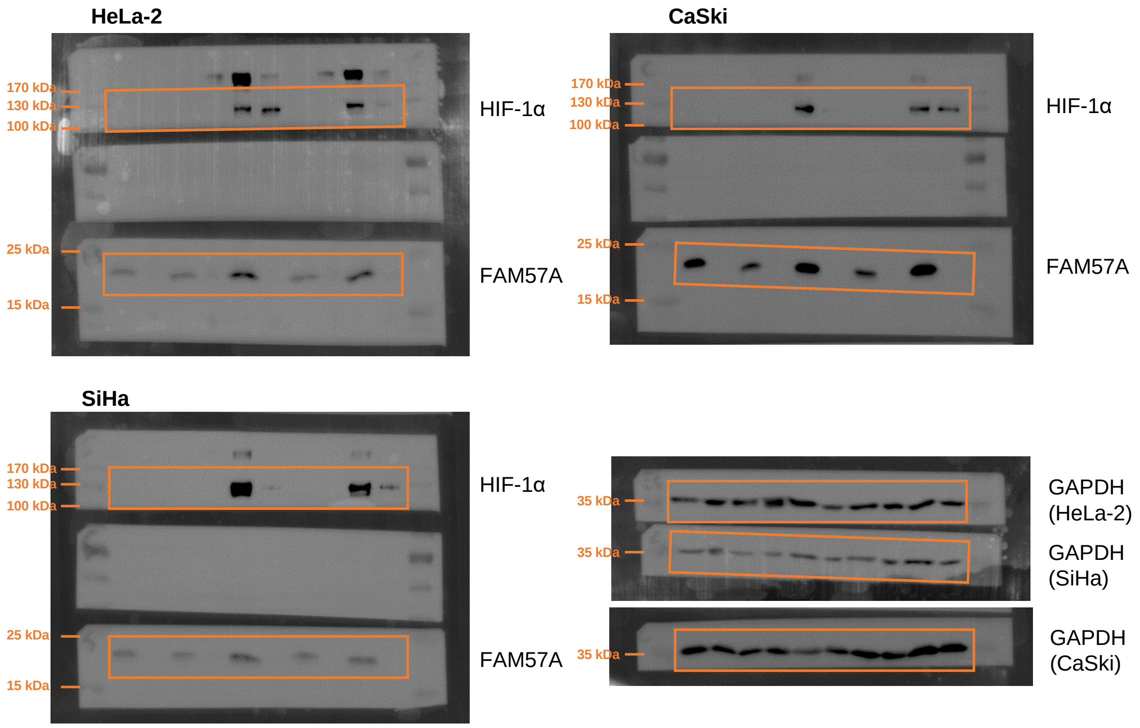


**HeLa-2**

| Lane | 1 | 2 | 3 | 4 | 5 | 6 | 7 | 8 | 9 | 10 | |
|---|---|---|---|---|---|---|---|---|---|---|---|
| **GAPDH** | 90.048 | 112.171 | 117.124 | 147.887 | 145.331 | 69.567 | 110.596 | 101.320 | 115.258 | 96.967 | raw values |
| **FAM57A** | 40.187 | 7.572 | 42.490 | 15.902 | 101.418 | 9.179 | 40.126 | 18.048 | 84.910 | 7.424 | raw values |
| | 1 | 0.151258 | 0.812885 | 0.240941 | 1.563671 | 0.295652 | 0.812971 | 0.399137 | 1.650731 | 0.171555 | relative values |
| **HIF-1α** | 0 | 0 | 0 | 0.106 | 146.507 | 146.956 | 0.293 | 0 | 145.272 | 27.781 | raw values |
| | 0 | 0 | 0 | 0.000711 | 1 | 2.095482 | 0.002628 | 0 | 1.25029 | 0.2842 | relative values |

**SiHa**

| Lane | 1 | 2 | 3 | 4 | 5 | 6 | 7 | 8 | 9 | 10 | |
|---|---|---|---|---|---|---|---|---|---|---|---|
| **GAPDH** | 114.796 | 130.396 | 80.879 | 83.362 | 147.175 | 91.941 | 138.362 | 120.125 | 169.358 | 110.679 | raw values |
| **FAM57A** | 42.502 | 3.864 | 30.499 | 7.544 | 74.465 | 8.348 | 41.439 | 0.726 | 57.117 | 1.077 | raw values |
| | 1 | 0.080037 | 1.018514 | 0.244428 | 1.366582 | 0.245240 | 0.808928 | 0.016324 | 0.910913 | 0.026283 | relative values |
| **HIF-1α** | 0.340 | 0 | 0 | 0.560 | 195.961 | 12.582 | 0.510 | 0 | 151.628 | 39.829 | raw values |
| | 0.002224 | 0 | 0 | 0.005045 | 1 | 0.102779 | 0.002768 | 0 | 0.672416 | 0.270270 | relative values |

**CaSki**

| Lane | 1 | 2 | 3 | 4 | 5 | 6 | 7 | 8 | 9 | 10 | |
|---|---|---|---|---|---|---|---|---|---|---|---|
| **GAPDH** | 149.955 | 133.417 | 121.25 | 127.968 | 100.747 | 126.298 | 154.201 | 144.259 | 182.193 | 177.150 | raw values |
| **FAM57A** | 136.289 | 2.136 | 82.829 | 2.733 | 165.96 | 1.497 | 102.789 | 2.587 | 189.389 | 1.143 | raw values |
| | 1 | 0.017615 | 0.751624 | 0.023498 | 1.812473 | 0.013041 | 0.733432 | 0.019731 | 1.143729 | 0.007099 | relative values |
| **HIF-1α** | 0 | 0 | 0 | 0.490 | 146.930 | 8.068 | 0 | 0 | 125.582 | 99.955 | raw values |
| | 0 | 0 | 0 | 0.002626 | 1 | 0.043802 | 0 | 0 | 0.472626 | 0.386888 | relative values |

**Figure S1** continued.

Related to Figure 4:

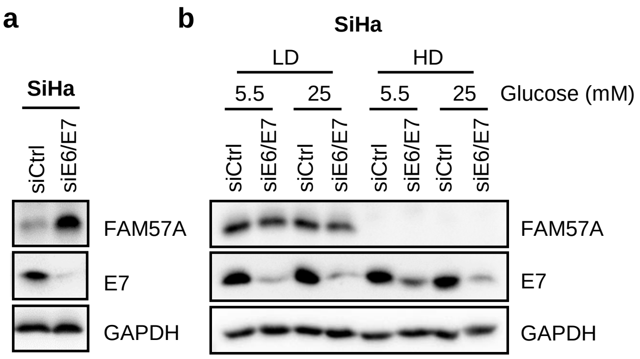


Original blots and quantification of blots corresponding to Figure 4:

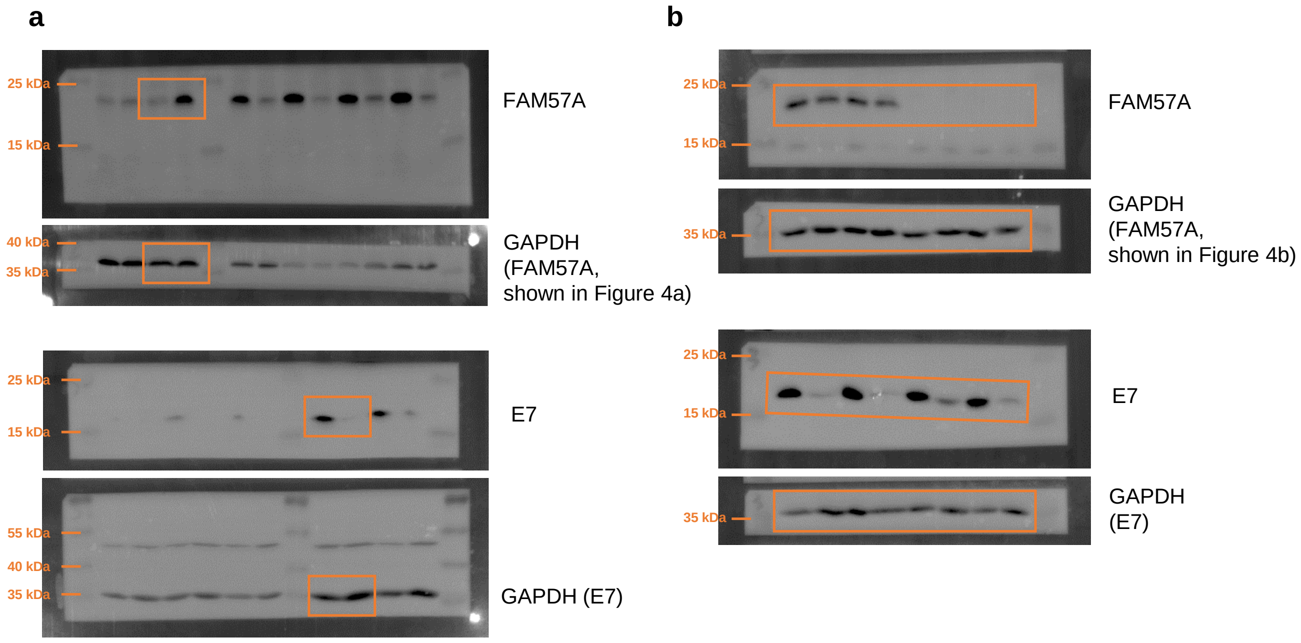


| Figure 4a | | | |
|---|---|---|---|
| Lane | 1 | 2 | |
| GAPDH (FAM57A) | 153.212 | 144.353 | raw values |
| FAM57A | 34.507 | 154.961 | raw values |
| | 1 | 4.766309 | relative values |
| GAPDH (E7) | 148.077 | 182.875 | raw values |
| E7 | 151.073 | 16.294 | raw values |
| | 1 | 0.087332 | relative values |

| Figure 4b | | | | | | | | | |
|---|---|---|---|---|---|---|---|---|---|
| Lane | 1 | 2 | 3 | 4 | 5 | 6 | 7 | 8 | |
| GAPDH (FAM57A) | 118.018 | 139.078 | 134.668 | 145.474 | 117.548 | 131.395 | 122.323 | 114.833 | raw values |
| FAM57A | 121.186 | 98.321 | 104.624 | 87.589 | 0 | 0 | 0.030 | 0 | raw values |
| | 1 | 0.688468 | 0.756594 | 0.586354 | 0 | 0 | 0.000239 | 0 | relative values |
| GAPDH (E7) | 127.043 | 176.172 | 179.62 | 156.905 | 153.151 | 166.974 | 131.019 | 156.848 | raw values |
| E7 | 144.869 | 28.366 | 146.299 | 29.385 | 140.862 | 68.000 | 137.888 | 32.128 | raw values |
| | 1 | 0.141201 | 0.714269 | 0.164234 | 0.806583 | 0.357137 | 0.922927 | 0.179630 | relative values |

**Figure S1** continued.

Related to Figure 6b:

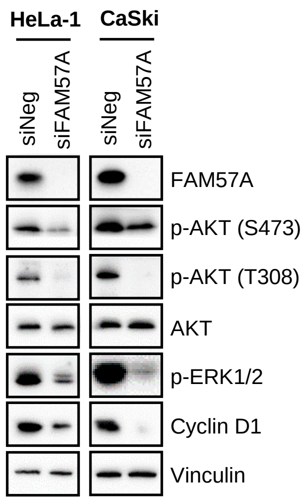


Original blots and quantification of blots corresponding to Figure 6b:

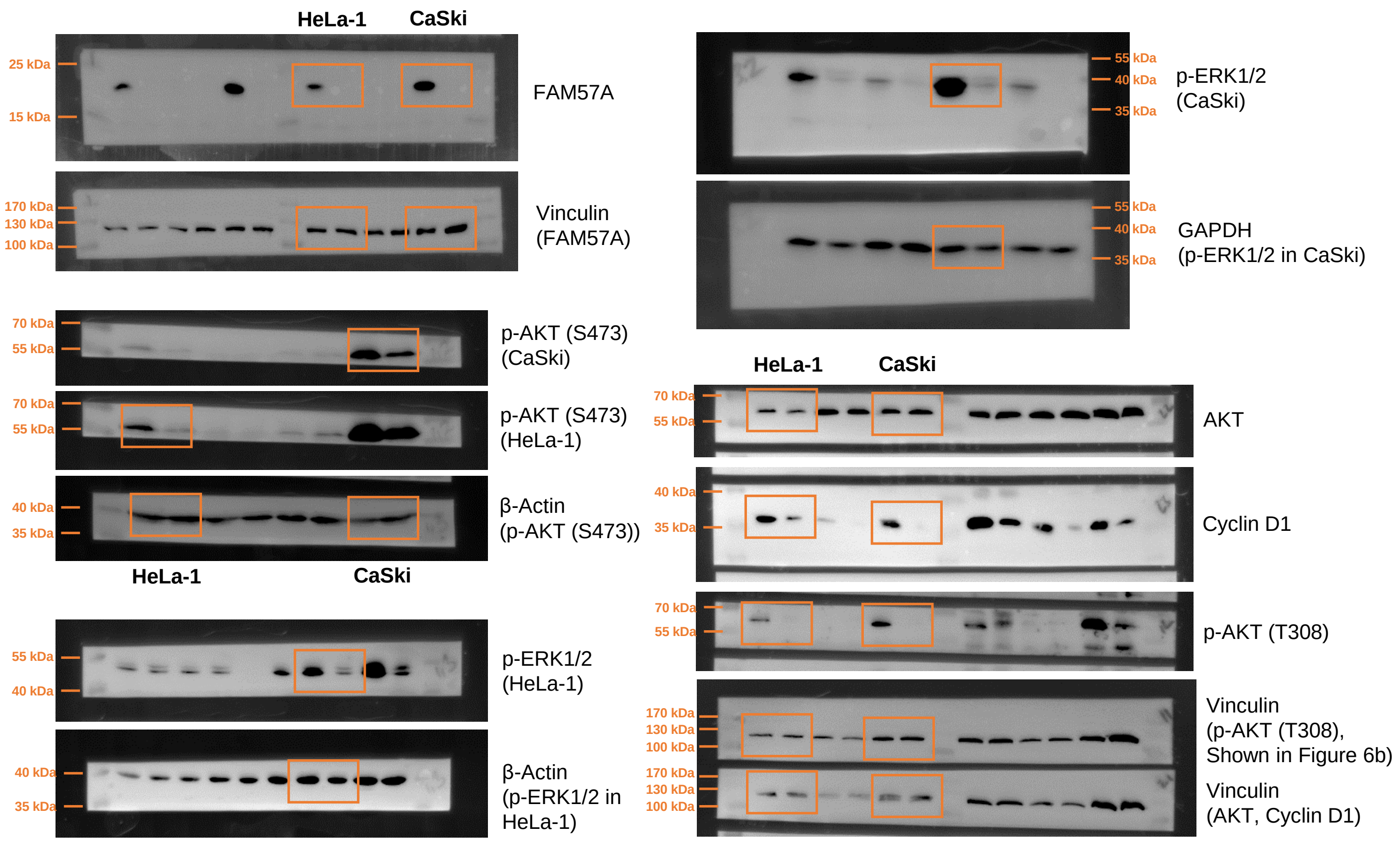


| | HeLa-1 | | CaSki | | |
|---|---|---|---|---|---|
| Lane | 1 | 2 | 1 | 2 | |
| **Vinculin (FAM57A)** | 110.536 | 120.756 | 115.962 | 158.590 | raw values |
| **FAM57A** | 152.229 | 3.286 | 179.214 | 1.758 | raw values |
| | 1 | 0.019759 | 1 | 0.007173 | relative values |
| **β-Actin (p-AKT (S473))** | 124.452 | 136.512 | 99.423 | 119.546 | raw values |
| **p-AKT (S473)** | 179.650 | 73.809 | 193.721 | 142.713 | raw values |
| | 1 | 0.374553 | 1 | 0.612687 | relative values |
| **Vinculin (p-AKT (T308))** | 131.134 | 132.705 | 164.795 | 150.136 | raw values |
| **p-AKT (T308)** | 1 | 0.087129 | 1 | 0.022291 | relative values |

| | HeLa-1 | | CaSki | | |
|---|---|---|---|---|---|
| Lane | 1 | 2 | 1 | 2 | |
| **Vinculin (AKT + Cyclin D1)** | 102.000 | 115.682 | 120.338 | 132.588 | raw values |
| **AKT** | 159.095 | 138.642 | 140.308 | 163.010 | raw values |
| | 1 | 0.768374 | 1 | 1.054461 | relative values |
| **Cyclin D1** | 170.545 | 71.645 | 171.416 | 11.416 | raw values |
| | 1 | 0.370409 | 1 | 0.060445 | relative values |
| **β-Actin/GAPDH (p-ERK1/2)** | 193.431 | 156.523 | 145.205 | 115.452 | raw values |
| **p-ERK1/2** | 119.040 | 25.503 | 172.531 | 12.628 | raw values |
| | 1 | 0.264756 | 1 | 0.092055 | relative values |

**Figure S1** continued.

Related to Figure S2:

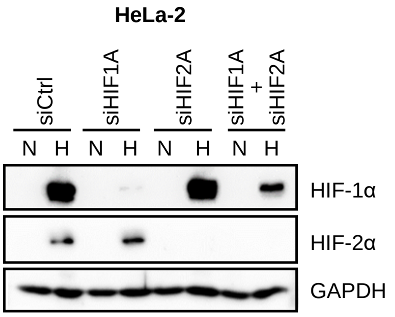


Original blots and quantification of blots corresponding to Figure S2:

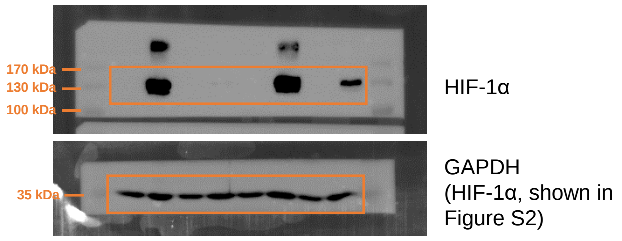


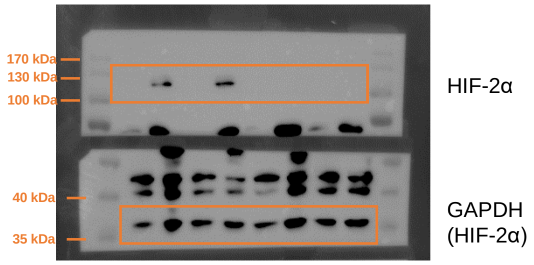


| | HeLa-2 | | | | | | | | |
|---|---|---|---|---|---|---|---|---|---|
| Lane | 1 | 2 | 3 | 4 | 5 | 6 | 7 | 8 | |
| **GAPDH (HIF-1α)** | 148.679 | **176.386** | 143.668 | 168.793 | 152.928 | 184.325 | 147.015 | 159.760 | raw values |
| **HIF-1α** | 2.147 | **196.033** | 1.473 | 5.310 | 0.382 | 207.190 | 1.944 | 92.412 | raw values |
| | 0.012993 | **1** | 0.009225 | 0.028306 | 0.002248 | 1.011392 | 0.011898 | 0.520469 | relative values |
| **GAPDH (HIF-2α)** | 93.614 | **157.210** | 125.762 | 128.547 | 106.816 | 174.701 | 129.632 | 156.884 | raw values |
| **HIF-2α** | 0.842 | **121.187** | 0.871 | 151.770 | 1.549 | 0.641 | 1.057 | 0 | raw values |
| | 0.011668 | **1** | 0.008984 | 1.53161 | 0.018812 | 0.00476 | 0.010578 | 0 | relative values |

**Figure S1** continued.

Related to Figure S3a:

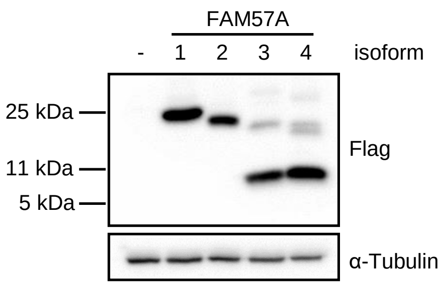


Original blots corresponding to Figure S3a (right panel):

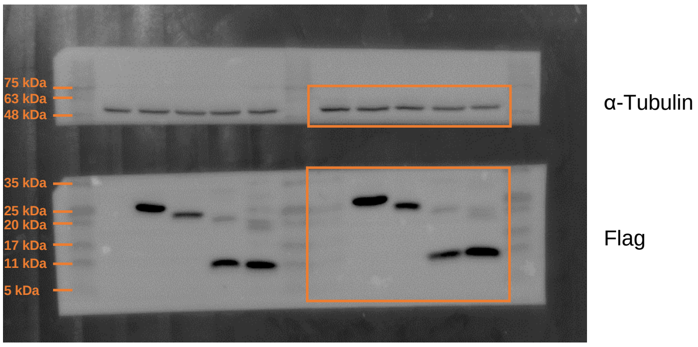


Related to Figure S3b:

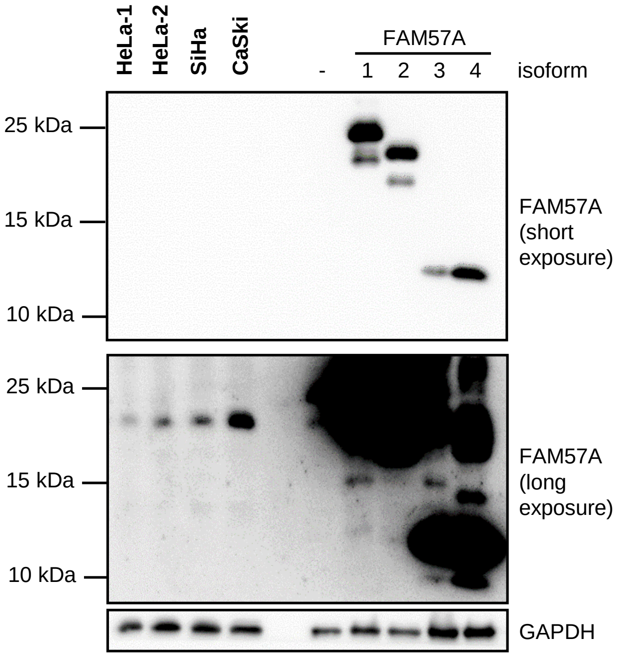


Original blots corresponding to Figure S3b (right panel):

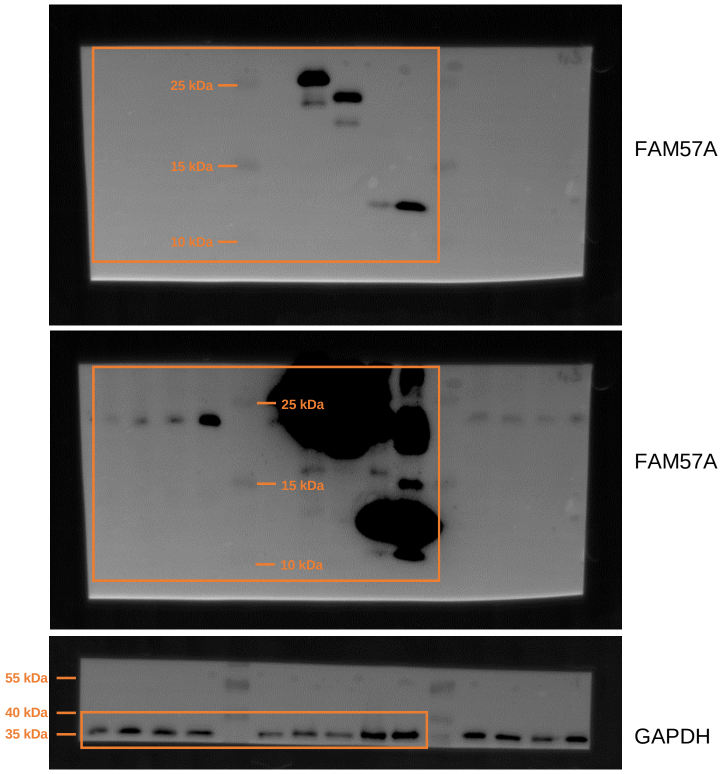


Related to Figure S3d:

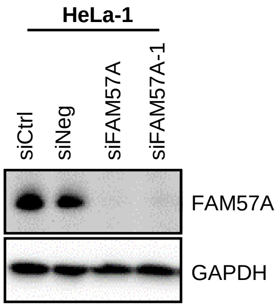


Original blots and quantification of blots corresponding to Figure S3d (right panel):

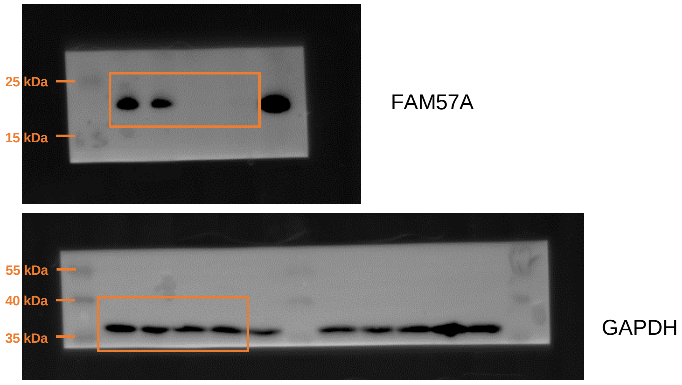


| | HeLa-1 | | | | |
|---|---|---|---|---|---|
| Lane | 1 | 2 | 3 | 4 | |
| **GAPDH** | 198.047 | 178.569 | 173.828 | 181.55 | raw values |
| **FAM57A** | 154.324 | 128.365 | 2.945 | 4.769 | raw values |
| | 1 | 0.922519 | 0.021742 | 0.033711 | relative values |

**Figure S1** continued.

Related to Figure S6c:

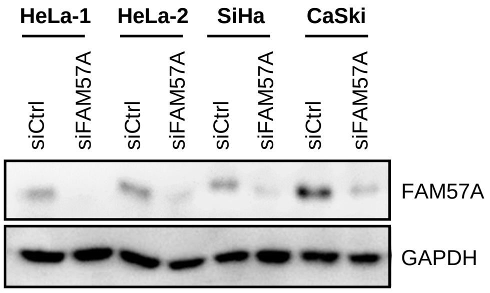


Original blots and quantification of blots corresponding to Figure S6c:

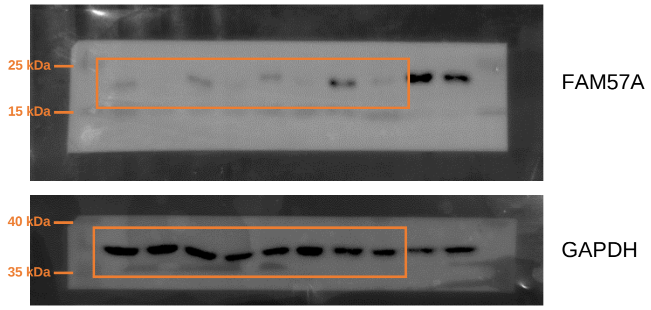


| | HeLa-1 | | HeLa-2 | | SiHa | | CaSki | | |
|---|---|---|---|---|---|---|---|---|---|
| Lane | 1 | 2 | 3 | 4 | 5 | 6 | 7 | 8 | |
| **GAPDH** | **99.200** | 129.217 | **113.500** | 92.683 | **139.182** | 132.067 | **128.550** | 98.267 | raw values |
| **FAM57A** | **30.874** | 10.081 | **83.852** | 14.437 | **38.443** | 8.261 | **43.594** | 14.183 | raw values |
| | **1** | 0.250670 | **1** | 0.210843 | **1** | 0.226467 | **1** | 0.425604 | relative values |

**Figure S1** continued.

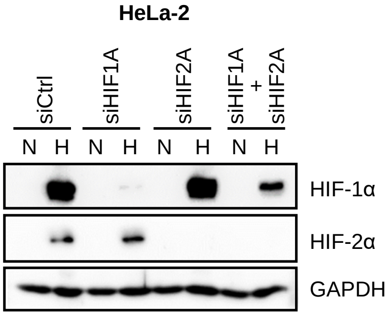


**Figure S2**. Immunoblot analyses measuring HIF-1α and HIF-2α protein levels following silencing *HIF1A* (siHIF1A) or *HIF2A* (siHIF2A) expression, either alone or in combination. siCtrl, control siRNA; GAPDH, loading control.

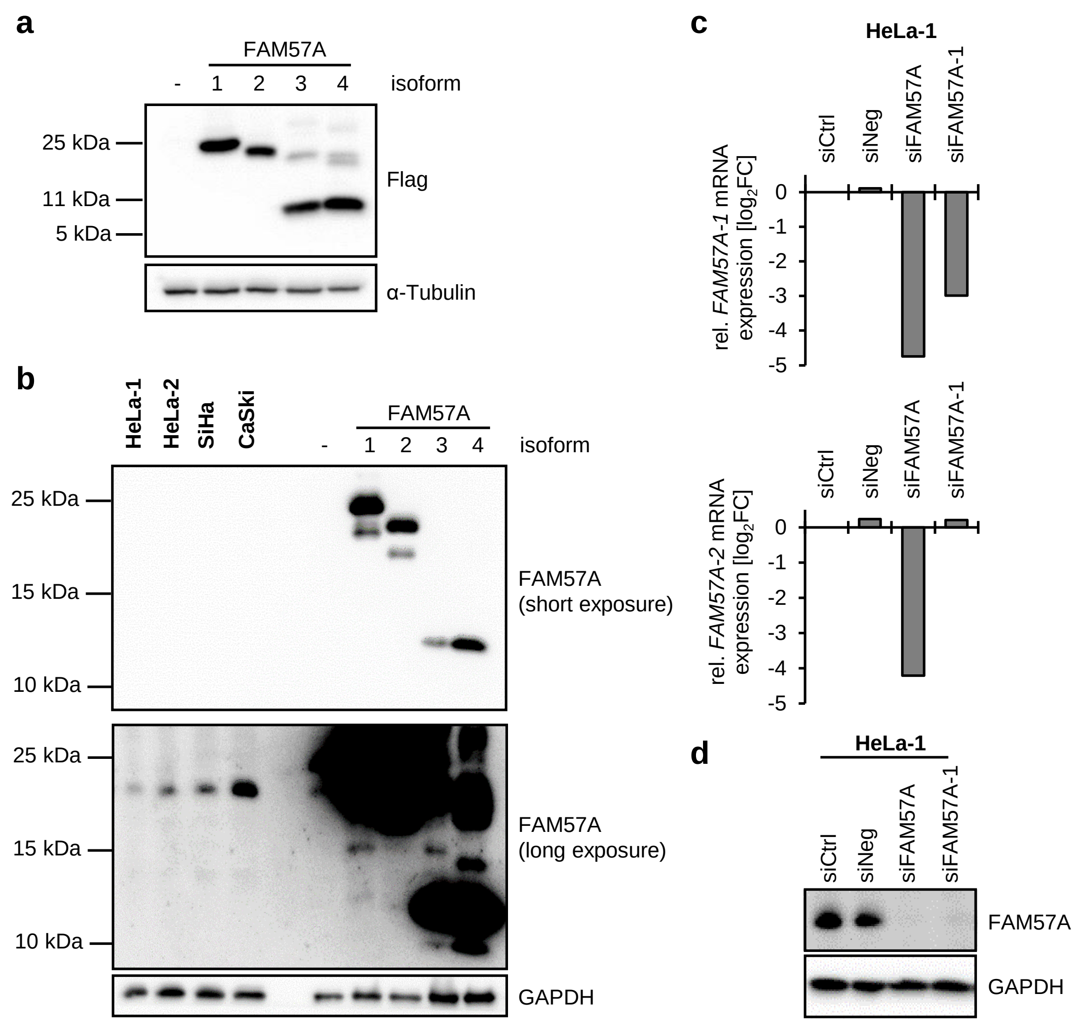


**Figure S3.** FAM57A antibody validation and detection of endogenous FAM57A-1 expression in cervical cancer cells. (**a**) The four predicted FAM57A isoforms (FAM57A-1, FAM57A-2, FAM57A-3, FAM57A-4) were ectopically overexpressed as Flag-tagged proteins in HeLa-1 cells and analyzed by immunoblot using an anti-Flag antibody. Size markers are indicated. α-Tubulin, loading control. (**b**) Endogenous FAM57A protein expression in HeLa-1, HeLa-2, SiHa and CaSki cells (left part of the figure) as well as ectopically overexpressed FAM57A in HeLa-1 cells (right part of the figure) were analyzed by immunoblot, employing the anti-FAM57A antibody generated in this work. Upper panel: short exposure, lower panel: long exposure of the same blot. Size markers are indicated. GAPDH, loading control. (**c, d**) In order to determine whether the endogenous FAM57A signal in HPV-positive cancer cells relates to the 257 amino acid FAM57A-1 (CT120A) or to the only 32 amino acid shorter FAM57A-2 (CT120B) isoform, we utilized an isoform-specific siRNA (siFAM57A-1). It efficiently downregulates the transcript coding for the FAM57A-1 isoform (upper panel), but does not affect the levels of the FAM57A-2-encoding transcript (lower panel) (**c**). Immunoblot analyses show that the endogenous FAM57A signal in HeLa-1 cells is efficiently downregulated by siFAM57A-1 (**d**), indicating that it is derived from the FAM57A-1 isoform. siCtrl and siNeg, control siRNAs; GAPDH, loading control.

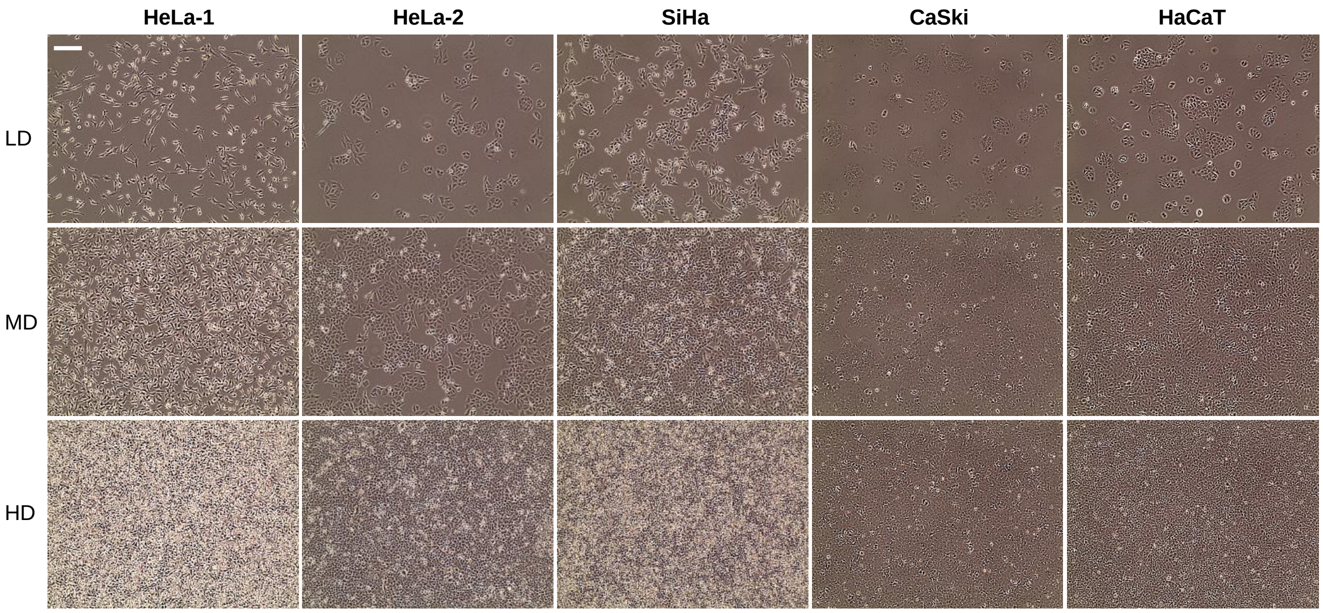


**Figure S4**. Microscopic visualization of different cell densities before harvesting the cells for measuring FAM57A expression levels by immunoblot analyses (relates to Figure 2). LD: low density; MD: medium density; HD: high density. A size marker is indicated in the upper left panel (scale bar: 200 µm).

5’-…CAGAGTCCTGCTCTGTGGCCCAGGCTGGAGTGCAGTG**GCGTG**ATCTCGGCTCACTGCAACCTCT
GCCTCCCAGGTTCAAGCGATTCTCCTGCCTCAGCCTCCAGAGTAGCTGGGACTACAGACAGGCACCAC
CACACCTGGCTAATTTTGTATTTTTAGTAGAGACGGGGTTTCACCGTGGTGGTCAGGCTGGTCTCGAAC
TCCTGACGTCAAGTGATCTGCCCGCCTCGGCCTCCCCAAGTGCTGGGGTTACAGGCGCGCCCGACCC
CGATACTTTTAAATAACGA**CACGT**TGTGAACAACACCACAGTCCCTACGCCTCTGGATGGTGTCTCCGG
ATAGATCCAAAGCCGAGAGGCAGTAAGAGCCTGCAGCCTCCACAGTCCGCCTCCTGGTGCAGGTGGA
GAGGGGCTGCTGGAGGCCCCCGGACCT**CACGC**TTCCTGAGGGCCACCCCGGAGCGGCTGCAGCCGC
TGGCGGCACCGGGAAAAGCATCTCGGCCGTGCGGGGAGAGGCCACATCCTGAGGCCTCCCACCGAGA
AGCACCAGGGCTTGGTGGACGGGGCCCGCGAACCCAGGGCCA**CACGC**CGCCTCCCTCCTTCCTTCCC
CGCGGGCCTCGCCCCACCCCGGAGGGGGTTCCTACCTGGCTTCCTCGGCCCCGCGCCCACCCCGCC
CCTTCGCCCGGGGCCGGCGAGAAGAGCAGGTCGGGGAGGGGCGCGTCCGCTGACGGAACAGAAGCC
GGGAGCCGCGGCCCTTCCCGCCTCCGCCTGGGCCCCGCGGCCGCTCGGGAGGGAGCGGCGGACTC
GGAACGCGCGCGCGCCGCGGCCCCGGCCACAGCCGCCGCCTCGGTCCCCGCCTCCGCCCCCGCCCA
CCCGCCCGCCGGGGACGCCGGGGAACCCTCCGGGCCCAGGGACCGCGAGAGACCCCGCCCCGCCC
TATCGCAGCCGCCGGGCCTGGT**CACGC**GCAGAGCCGCGCCGCGGGATCGGGGCCAGCGCCGGGCG
GAGGCGGGACGGGGACGGGGGCGGAGGGTTGAAATCGCGCGGCCGGGCCGGGGCGCGCC***G***AGCC
GAACCCAGC**CACGC**GGCGCCAGCGAGGCGGCCGGACCCGCAGCCCCGATGCTGCTGACG…-3’

**Figure S5**. Potential HIF-1 binding sites in the promoter region (nucleotides -1056 to +64) of the *FAM57A* gene. Core consensus binding sequences for HIF-1 (5’- A/G-C-G-T-G -3’ or its complementary sequence 5’- C-A-C-G-T/C -3’) are highlighted in bold. The predicted transcriptional start site is marked in bold and italicized, the underlined sequence corresponds to the start of the coding sequence [9].

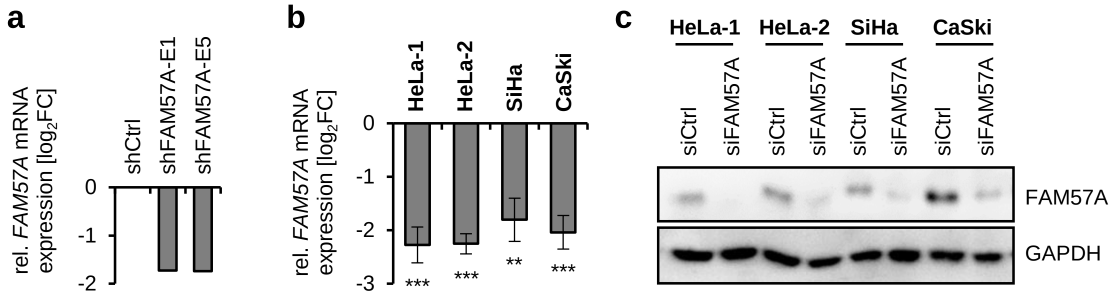


**Figure S6**. Validation of shRNAs and siRNAs used for *FAM57A* silencing. (**a**) qRT-PCR analyses of *FAM57A* transcript levels upon transfection of HeLa-1 cells with pSUPER plasmids expressing two different *FAM57A*-targeting shRNAs (shFAM57A-E1 or shFAM57A-E5). shCtrl: control shRNA. Shown are the $log_2FC$ of the *FAM57A* levels following *FAM57A* silencing relative to shCtrl-expressing cells ($log_2FC=0$). (**b**) Validation of siFAM57A (equimolar pool of siFAM57A-E1 and siFAM57A-E5) in HPV-positive cancer cell lines at the transcript level by qRT-PCR. Shown are the $log_2FC$ values of mean *FAM57A* transcript levels of siFAM57A-transfected cells relative to control siRNA (siCtrl)-transfected cells ($log_2FC=0$) with standard deviations from at least 3 independent experiments for each cell line. Statistically significant differences to the respective siCtrl-transfected cells are determined by one-way ANOVA, **: $P < 0.01$, ***: $P < 0.001$. (**c**) Corresponding analyses of FAM57A protein levels by immunoblot analysis under the treatment conditions outlined in subfigure (b). siCtrl, control siRNA; GAPDH, loading control.
